# Acteoside relieves diabetic retinopathy through the inhibition of Müller cell reactive hyperplasia by regulating TXNIP and mediating Kir4.1 channels in a PI3K/Akt-dependent manner

**DOI:** 10.1371/journal.pone.0312565

**Published:** 2024-12-17

**Authors:** Xiaoting Xi, Xiaolei Liu, Qianbo Chen, Jia Ma, Xuewei Wang, Yufei Gui, Yuxin Zhang, Yan Li

**Affiliations:** 1 Ophthalmology Department, The First Affiliated Hospital of Kunming Medical University, Kunming, China; 2 Neurology Department, The First Affiliated Hospital of Kunming Medical University, Kunming, China; New York University Langone Health, UNITED STATES OF AMERICA

## Abstract

Diabetic retinopathy (DR) is a severe microangiopathy of diabetes. Müller cells play an important role in the development of DR. Acteoside (ACT) has been reported to be effective in the treatment of DR. In this study, we explored the molecular mechanism of ACT in the treatment of DR from the perspective of the reactive proliferation of Müller cells. The effect of ACT on DR was investigated via high-glucose (HG) treatment of Müller RMC-1 cells and an injection of streptozotocin (STZ) in constructed DR cells and animal models. The results showed that after ACT treatment, damage to the retinal structure in DR rats was alleviated, the number of hemangiomas was reduced, and the penetration of blood vessels was weakened. In addition, ACT treatment improved the hypertrophy and gliogenesis of Müller cells during DR, promoted the expression of Kir4.1 and activated the PI3K/Akt signaling pathway. ACT treatment inhibited the proliferation and migration of RMC-1 cells and promoted the expression of Kir4.1. TXNIP overexpression effectively reversed the inhibitory effect of ACT on the proliferation and migration of Müller cells and its induction of Kir4.1 expression. In addition, TXNIP knockdown effectively reversed the inhibitory effect of HG on the expression of p-PI3K and p-Akt, whereas TXNIP overexpression had the opposite effect, and treatment with the PI3K/AKT pathway inhibitor LY294002 effectively reversed the effect of TXNIP knockdown. Animal experiments also confirmed that the therapeutic effect of ACT on DR rats could be reversed by the overexpression of TXNIP or LY294002. In conclusion, ACT inhibits Müller cell reactive proliferation and alleviates diabetic retinopathy by regulating TXNIP and mediating the expression of Kir4.1 channels in a PI3K/Akt-dependent manner.

## 1 Introduction

Diabetic retinopathy (DR) is a common ocular microvascular complication of diabetes and a major factor contributing to blindness and visual impairment [1]. In recent years, the incidence of diabetes has been increasing, and the associated burden of this disease has increased significantly [2]. In addition, patients can develop different degrees of vision problems, such as blurred vision, vitreous hemorrhage, retinal detachment and even blindness [3]. The substantial negative impacts of DR on human health and quality of life cannot be ignored. At present, laser photocoagulation, glucocorticoids, vitrectomy, and neutralizing antivascular endothelial growth factor (VEGF) drugs, which are relatively mature DR treatments, have shown some clinical benefits, but complete control of the disease is still insufficient [4, 5]. Ideas for new drugs targeting other non-VEGF-driven pathways or novel therapeutic strategies for DR gene therapy have also been proposed [6]. Therefore, the study of the nonvascular pathological mechanisms of DR is an important breakthrough direction for new treatment methods.

Acteoside (ACT) is a phenylpropanoside isolated from and widely distributed in dicotyledonous plants, and more than 150 types of plants containing ACT have been identified, such as *Scrophularia ningpoensis*, *Cistanche deserticola*, and *Digitalis purpurea* [7]. ACT has been shown to have a variety of pharmacological activities, including antidiabetic effects [8]. For example, studies have shown that ACT can alleviate retinal damage in diabetic mice by inhibiting oxidative stress-induced damage of retinal pigment epithelial (RPE) cells [9]. ACT also improves kidney injury in diabetic mice [10]. However, very few studies have examined the role of ACT in the treatment of DR, and many gaps remain to be explored. Müller cells are the most important glial cells in the retina and play an important role in maintaining the morphological structure of the blood‒retinal barrier (BRB) [11]. Reactive gliosis of Müller cells is an early feature of DR and a hallmark of retinal damage [12]. In addition, hypertrophy (increase in cell volume) and hyperplasia (increase in the number of cells) are features of Müller cell reactive gliosis [13]. Kir4.1 is the main protein responsible for drainage in Müller cells, and the coexpression of Kir4.1 and aquaporin 4 (AQP4) in retinal Müller cells tightly regulates retinal water homeostasis. Müller cells can also maintain retinal K^+^ concentrations through Kir4.1 channels [14, 15]. During DR, the expression of Kir4.1 in Müller cells decreases, which can lead to Müller cell swelling or retinal vascular leakage [16]. Studies have also shown that after DR rats are treated with the potassium channel opener pinacidil, Kir4.1 expression is increased and the function of Müller cells is improved, thereby reducing the symptoms of macular edema [17]. In summary, we propose an interesting hypothesis that ACT is able to alleviate the progression of DR by inhibiting the reactive proliferation of Müller cells via the Kir4.1 channel. Based on this conjecture, the molecular regulatory mechanism was further explored.

One study showed that Kir4. channels may be revitalized by insulin and insulin-like growth factor-1 in a PI3K-dependent manner [18]. In addition, studies have shown that inactivation of Kir4.1 can reduce the activity of the PI3K/AKT pathway, thereby triggering cells to enter the resting state [19]. In addition, after an ischemic attack, Kir4.1 can be revitalized in a PI3K-dependent manner, increasing cell survival via the mTOR pathway [20]. These findings fully illustrate the close relationship between PI3K/AKT and Kir4.1, and this relationship is important and dependent on Kir4.1 channel activation. Previous studies have shown that ACT can alleviate oxidative stress in retinal ganglion cells (RGCs) by revitalizing the PI3K/AKT pathway [21]. These findings led us to speculate that ACT can mediate Kir4.1 channel expression through the regulation and activation of PI3K/Akt. In addition, a study revealed that TXNIP could regulate autophagy and apoptosis in Müller cells in rats with DR by restraining the PI3K/AKT/mTOR pathway [22]. We speculate that ACT mediates Kir4.1 channel expression in a PI3K/Akt-dependent manner by regulating TXNIP.

In summary, this study proposed that ACT inhibits Müller cell reactive hyperplasia by regulating TXNIP and mediating Kir4.1 channel expression in a PI3K/Akt-dependent manner to ameliorate DR, and we further investigated this hypothesis using cell and animal experiments. The specific molecular mechanisms underlying the treatment of DR by ACT were eventually elucidated, which provided potential cellular and therapeutic targets for new treatment methods for DR.

## 2 Materials and methods

### 2.1 Cell culture and treatment

Retinal Müller RMC-1 cells (HTX2216) were purchased from OTWO Biotech. RMC-1 cells were cultured in DMEM supplemented with 10% FBS (Gibco, USA) and 1% penicillin and streptomycin (Gibco, USA). The incubator conditions were 37°C and 5% CO_2_. RMC-1 cells were subjected to the following different treatments: osmotic pressure control (Mannitol) group—RMC-1 cells were treated with D-glucose (5.5 mmol/L) + mannitol (24.5 mol/L); normal glucose (NG) group—RMC-1 cells were treated with 5.5 mmol/L D-glucose; high glucose (HG) group—RMC-1 cells were treated with 33 mmol/L D-glucose; and ACT treatment (HG+ACT) group—HG RMC-1 cells were treated with 30 μM ACT (HY-N0021, MedChem Express, Monmouth Junction, USA) [9]. In addition, RMC-1 cells were treated with the PI3K/Akt inhibitor LY294002 (10 μM) [23] or the PI3K/Akt activator IGF-1 (10 nM) [24].

### 2.2 Cell transfection

RMC-1 cells were seeded in 6-well plates. The pcDNA3.1 vector was used to construct the TXNIP overexpression plasmid (pc-TXNIP). sh-TXNIP was obtained from GenePharma Co., Ltd. pc-TXNIP and sh-TXNIP were transfected into RMC-1 cells according to the specifications of the Lipofectamine 2000 Transfection Kit (Invitrogen, USA). Forty-eight hours after transfection, the transfection efficiency was detected or subsequent experiments were performed.

### 2.3 Construction of the rat DR model

Male rats were obtained from Hunan Slake Jingda Experimental Animal Co., Ltd. SPF rats are housed at a temperature of 20~25°C with a humidity of 50%~65%. After the rats were fasted overnight, the rats were anesthetized with isoflurane and intraperitoneally injected with 60 mg/kg STZ [25]. The injection was continued for 5 days to induce the diabetes model. After 48 hours, the fasting blood glucose levels of the rats were measured, and when the blood glucose concentration was greater than 16.7 mmol/L for 5 consecutive days, the diabetic rat model was successfully established. After the model was successfully established, 5 μL of different concentrations of ACT (1 mg/ml, ACT-L; 2 mg/ml, ACT-M; or 5 mg/ml, ACT-H) solutions was injected intravitreally. Alternatively, adenovirus containing the pc-TXNIP plasmid and LY294002 were injected into the tail vein. After the experimental observations, the rats were sacrificed via cervical dislocation. The animals were observed for pain during the experiment, and if more than expected and transient signs of pain or distress were observed, the rats were administered analgesics or a placebo. All animal experimental protocols were approved by the Ethics Review Committee of Kunming Medical University (kmmu20211334). All methods were performed in accordance with the relevant guidelines and regulations and the ARRIVE guidelines.

### 2.4 HE staining

After the rat eyeball tissue was embedded in paraffin and cut into thin sections, it was dewaxed and hydrated with xylene I (10 min), xylene II (10 min) and different concentrations of alcohol (100%, 95%, 85%, 75% alcohol, respectively for 2 min). Then, the sections were soaked in hematoxylin for 3–5 min, flushed with tap water, differentiated for 5–10 s in 1% hydrochloric acid in alcohol, the color was reversed to blue for 10 min in tap water, and sections were stained with eosin for 10 s. After staining with eosin, the samples were dehydrated with different concentrations of alcohol and then treated with xylene I and xylene II. After drying, the sections were mounted with neutral gum. Finally, the sections were viewed and photographed under a light microscope.

### 2.5 Retinal mounts and fluorescence staining

After the rat eyeball was removed, a 1–3 mm puncture incision was made at the corneal scleral limbus. The eyeballs were then fixed with 4% paraformaldehyde for 1 h and rinsed with PBS. Under a dissecting microscope, the eyeball was cut along the cornea of the eyeball, the lens was removed, the sclera was cut to peel off the retina, and the detached retina was laid flat on a glass slide. The whole mounts were incubated with PBS after baking in a 37°C oven and then fluorescently stained with condensin B4-FITC (10 μg/mL). The samples were placed in a humid box in a 4°C refrigerator and incubated overnight. After the samples were rinsed with PBS, they were observed and photographed under a fluorescence microscope.

### 2.6 Staining with Evans blue dye

Evans blue dye (30 mg/mL) was dissolved in normal saline and injected into the rats via the tail vein. The staining was successful when the rat eye turned blue. After 2 hours, the eyeballs were removed for retinal mounting, the images were viewed under a fluorescence microscope with a red filter (BX51, Olympus Optical Co. Ltd. Tokyo, Japan), and the morphology and leakage of the rat retina were observed.

### 2.7 RT‒qPCR

Total RNA was extracted from cells and tissues using TRIzol reagent. The RNA was reverse transcribed into cDNA using a reverse transcription kit. Real-time PCR was performed with the SYBR Green Real-Time PCR Kit using cDNA as a template. GAPDH was used as the internal control, and the results were calculated using the 2^−ΔΔCt^ method. Details of the primer sequences are shown in Table 1.

**Table 1:** Primer sequences.

Gene	Sequence (F: Forward primer; R: Reversed primer)
*TXNIP*	F: 5´-TCCGAGTGCAGAAGATCAG-3´
	R: 5´-CACTAACATAGATCAGCAAGGAG-3´
*GFAP*	F: 5´-AAATCTGTGTCAGAAGGCCA-3´
	R: 5´-CTCCTTAATGACCTCGCCA-3´
*Kir4*.*1*	F: 5´-CACTTACTGGGCCTTCCTC-3´
	R: 5´-AATGAGAAGCACAATGGCC-3´
*GAPDH*	F: 5´-AACTCCCATTCTTCCACCT-3´
	R: 5´-TTGTCATACCAGGAAATGAGC-3´

### 2.8 Western blot

Total protein was extracted from cells and tissues using RIPA buffer (Sigma Aldrich, Cambridge, MA), and the protein concentration was measured with a BCA kit (Sigma Aldrich, Cambridge, MA). The samples were collected and loaded, and the proteins were separated on 10% SDS‒PAGE gels and transferred to PVDF membranes that were subsequently blocked with 5% skim milk for 1 h. Primary antibodies against TXNIP(ab188865, 1:1000, Abcam, UK), PI3K (MA1-74183, 1: 2000, Invitrogen, USA), p-PI3K (PA5-17387, 1:1000, Invitrogen, USA), AKT (ab8805, 1: 1000, Abcam, UK), p-AKT (ab38449, 1:1000, Abcam, UK), GFAP(ab302644, 1:1000, Abcam, UK), Vimentin (ab20346, 1:1000, Abcam, UK), Nestin (ab105389, 1:100, Abcam, UK), Kir4.1(ab306550, 1:1000, Abcam, UK), Na^+^-K^+^-ATPase(Cat. 3010S, 1:1000, Cell Signaling Technology, USA), GAPDH(ab8245, 1:1000, Abcam, UK) were added, and the samples were incubated at 4°C overnight. The next day, the membrane was incubated with the corresponding secondary antibody for 1 h at room temperature. The protein bands were visualized using an enhanced chemiluminescence (ECL) kit (Millipore, USA) and a gel imaging system (Thermo Fisher, USA). Finally, The gray values of protein bands were analyzed using GAPDH as internal referenceand.

### 2.9 Observation of semithin retinal sections

Rat eyeballs were fixed with 2.5% glutaraldehyde for 30 min. The anterior part of the eye was excised under a dissecting microscope, and the remaining posterior part was fixed with 2.5% glutaraldehyde for 5 h. The tissues were dehydrated with various concentrations of ethanol and embedded in epoxy resin for sectioning. The sections were dyed with toluidine blue (1 μM) and viewed. The morphology of the cells was observed under an optical microscope.

### 2.10 CCK-8

RMC-1 cells were seeded in 96-well plates at a density of 1 × 10^5^ cells/well. After an incubation for 24 h at 37°C with 5% CO_2_, 10 μL of CCK-8 solution (Solarbio, Beijing, China) was added to each well. After an incubation for 2 h, the absorbance at 450 nm was measured with a microplate reader.

### 2.11 EdU

An EdU cell proliferation kit (ab219801, Abcam, UK) was used to detect cell proliferation. Briefly, RMC-1 cells were first cultured in 200 μL of medium. When the cells reached 60–70% confluence, 100 μL of cell culture medium mixed with 100 μL of 2× EdU solution was incubated with the cells for 3 h. Afterward, 200 μL of 1× fixative solution was added to each well, and the mixture was allowed to incubate for 15 min at room temperature in the dark. After the fixative mixture was aspirated, the wells were flushed with wash buffer, 200 μL of 1× permeabilization buffer was added to each well, and the mixture was incubated at room temperature for 20 min. The reaction mixture was added according to the instructions to complete the EdU reaction. Then, 100 μL of DAPI working solution was added to the cells, which were incubated for 30 min for nuclear staining. After the cells were flushed with PBS, they were observed under a fluorescence microscope.

### 2.12 Transwell

After RMC-1 cells were digested with trypsin, they were flushed twice with PBS and resuspended in serum-free medium containing BSA to adjust the cell density to 5 × 10^5^/ml. After the Transwell insert was placed in the culture plate, 200 μL of cell suspension was added to the upper chamber, and 600 μL of substrate containing 20% serum was added to the lower chamber. After culture for 24 h, the medium was discarded, the cells were flushed with PBS, and the cells were fixed with methanol for 30 minutes. After the wells were air-dried, the cells were dyed with 0.1% crystal violet for 20 min. The unmigrated cells in the upper layer were removed with cotton swabs and the insert was flushed 3 times with PBS. Finally, the cells were observed and counted under a microscope.

### 2.13 Cell scratches

Single-cell suspensions were prepared from RMC-1 cells in the logarithmic growth phase using trypsin and seeded into 6-well plates. The cells were cultured overnight and scratched with a 200 μL pipette tip when the confluence reached 100%. Then, the cells were flushed with PBS to eliminate the scratched cells, serum-free medium was added, and the cells were placed in a 37°C, 5% CO_2_ incubator for culture. Afterward, images were captured with a microscope at 0 h and 24 h and analyzed via ImageJ software. The cell mobility rate (wound healing rate) was calculated as follows: (area of scratches at 0 h—area of scratches at 24 h)/area of scratches at 0 h.

### 2.14 Statistical analysis

GraphPad Prism 7.0 was used to analyze the experimental data and plot the graphs. All the cell experiments were repeated 3 times, all the animal experiments were repeated 5 times, and the experimental data are presented as the means ± standard deviations (means ± SD). Comparisons between two groups were performed using Student’s *t* test, and comparisons among multiple groups were performed using one-way or two-way ANOVA. P < 0.05 indicated a statistically significant difference.

## 3 Results

### 3.1 ACT treatment relieves DR in rats

**Fig 1:**
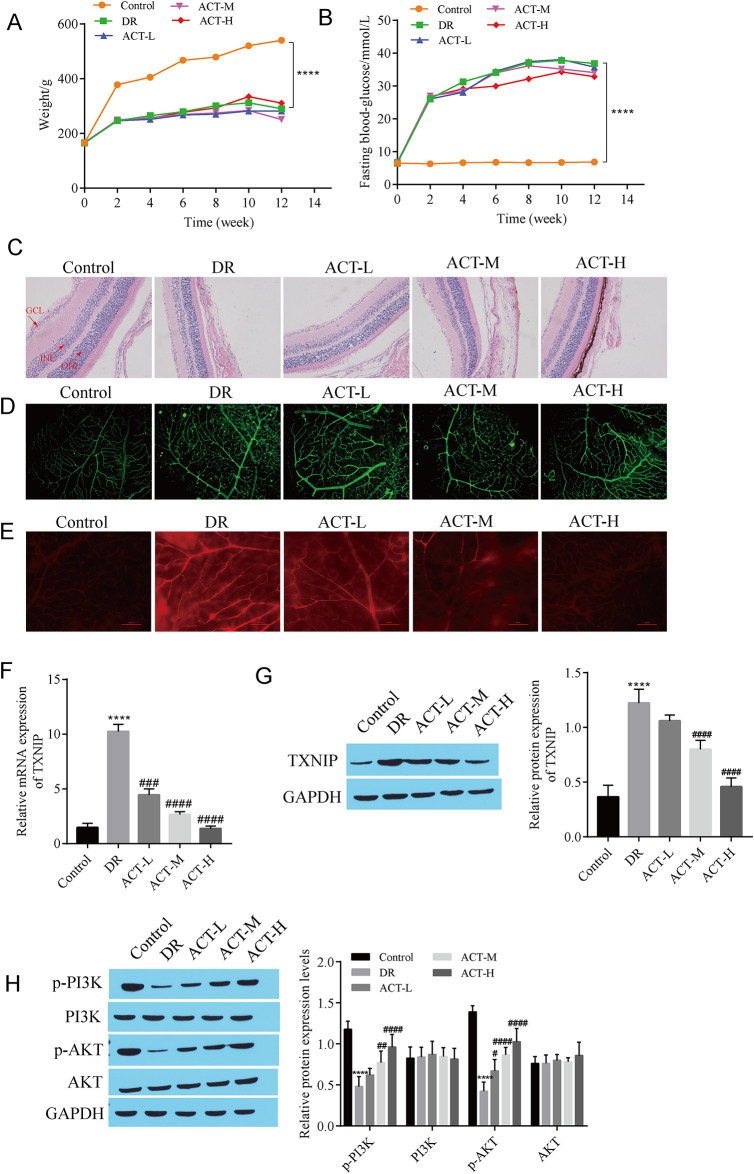
ACT treatment relieves DR in rats. A: Alterations in rat body weight (0, 2, 4, 6, 8, 10, and 12 w). B: Changes in fasting blood glucose levels (mmol/L) in rats (0, 2, 4, 6, 8, 10, and 12 w). C: Changes in the retinal morphological structure observed with HE staining. D: The morphology of retinal blood vessels was observed by retinal smearing and fluorescent staining. E: Evans blue staining to detect the permeability of retinal blood vessels. F: RT‒qPCR analysis of *TXNIP* expression. G: Western blot showing TXNIP expression. H: Western blots showing the levels of p-PI3K, PI3K, p-AKT, and AKT. After the DR Rats were constructed by intraperitoneal injection of STZ (60 mg/kg), the rats were treated with different concentrations of ACT(1 mg/ml, ACT-L; 2 mg/ml, ACT-M; or 5 mg/ml, ACT-H), and the body weight and fasting blood glucose of the rats were monitored at week 0, 2, 4, 6, 8, 10 and 12. After the monitoring, the rats were killed and the eyeball tissues were taken to detect various indexes. ****P<0.0001, compared with the control group; ^#^P<0.05, ^##^P<0.01, ^###^P<0.001, and ^####^P<0.0001, compared with the DR group.

The rats were peritoneally injected with STZ to establish an animal model of diabetic retinopathy (DR), and the rats were treated with different concentrations of acteoside (ACT). The body weights and blood glucose concentrations of the rats were monitored every 2 weeks after injection. The results revealed no significant difference in body weight or blood glucose concentrations between the groups at 0 weeks. Compared with those in the control group, the body weights of the rats in the DR, ACT-L, ACT-M and ACT-H groups were significantly reduced, and the blood glucose concentration increased (Fig 1A and 1B). However, no prominent differences in rat body weight or blood glucose concentrations were observed among the DR group, the ACT-L group, the ACT-M group, or the ACT-H group (Fig 1A and 1B). HE staining was used to observe the morphological and structural changes in the retina. The results revealed that the retinal structure of the control group was clear and complete, while the retinal structure of the DR group was damaged, the number of cells in the ganglion cell layer (GCL) and the nuclear morphology were abnormal, and the nuclei of the inner core layer (INL) and outer nuclear layer (ONL) were swollen and disordered, but the damage to the retinal structure was alleviated to a certain extent in the ACT treatment group (Fig 1C). Retinal placement and fluorescence staining revealed that the retinal blood vessels in the control group were radial, the blood vessel wall was smooth, and no microangioma occurred; however, clustered microangiomas were observed around the retinal blood vessels in the DR group, and the number of hemangiomas in the ACT treatment group decreased (Fig 1D). Evans blue dye was used to detect the permeability of retinal blood vessels, and the results revealed that the permeability of retinal blood vessels was significantly greater in the DR group than in the control group, and this phenomenon was alleviated after ACT treatment (Fig 1E). RT‒qPCR and Western blotting were used to detect TXNIP mRNA and protein expression, respectively. The results revealed that TXNIP expression was significantly upregulated in the DR group compared with that in the control group, TXNIP expression was downregulated in the ACT group compared with that in the DR group, and the downregulation was greatest in the ACT-H group (Fig 1F and 1G). Finally, Western blot analysis of the levels of proteins in the PI3K/AKT signaling pathway revealed that, compared with those in the control group, the expression of p-PI3K and p-AKT in the DR group was downregulated, and the expression of p-PI3K and p-AKT gradually increased after treatment with increasing ACT concentrations, while no significant difference in the expression of PI3K and AKT was observed among the groups (Fig 1H). These results suggest that ACT can alleviate diabetic retinopathy in rats in a concentration-dependent manner, and its effect may be related to the TXNIP and PI3K/Akt signaling pathways.

### 3.2 ACT improves the process of DR-induced pathological changes in Müller cells

**Fig 2:**
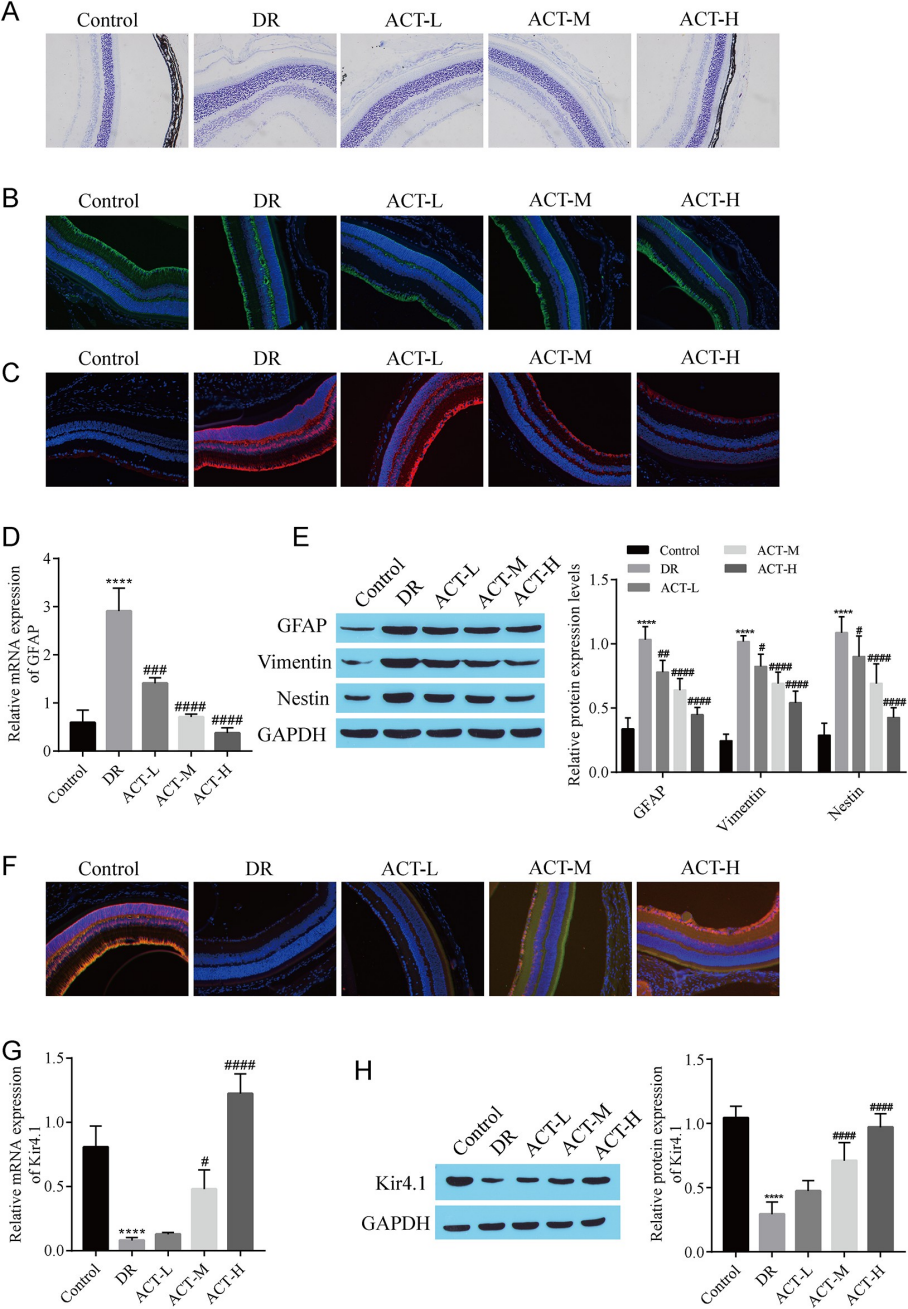
ACT improves the process of DR-induced pathological changes in Müller cells. A: Müller cells observed in semithin sections of the retina. B: Immunofluorescence detection of Müller cells expressing the cell-specific marker CRALBP. C: GFAP expression detected using immunofluorescence staining. D: RT‒qPCR detection of *GFAP* expression. E: Western blots showing the levels of GFAP, Vimentin, and Nestin. F: Double immunofluorescence staining for the expression of Kir4.1 and GS. G: RT‒qPCR analysis of *Kir4*.*1* levels. H: Western blot showing Kir4.1 expression. After the DR Rats were constructed by intraperitoneal injection of STZ (60 mg/kg), the rats were treated with different concentrations of ACT(1 mg/ml, ACT-L; 2 mg/ml, ACT-M; or 5 mg/ml, ACT-H). After the monitoring, the rats were killed and the eyeball tissues were taken to detect various indexes. ****P<0.0001, compared with the control group; ^#^P<0.05, ^##^P<0.01, ^###^P<0.001, and ^####^P<0.0001, compared with the DR group.

Müller cells play important roles in DR development. Therefore, we investigated the influence of ACT on pathological changes in Müller cells during DR to clarify the specific mechanism of the effect of ACT. Semithin sections of the retina revealed fluid accumulation between the nuclei of the outer nuclear layer of the DR group as banded clear gaps, indicating an enlarged apical process of Müller cells, whereas treatment with ACT reduced the number of banded clear spaces in the retina (Fig 2A). Immunofluorescence staining was performed to detect the expression of cellular retinaldehyde-binding protein (CRALBP), a marker specifically expressed by Müller cells. The results revealed that the Müller cells in each group expressed CRALBP with no significant difference in expression (Fig 2B), but glial fibrillary acidic protein (GFAP), a sensitive marker of Müller cell reactive hyperplasia, was expressed only in astrocytes located in the nerve fiber layer in the control group, whereas the area of GFAP expression was increased in the DR group, and the comprehensive expression of GFAP was inhibited to a certain extent by ACT treatment (Fig 2C). RT‒qPCR also revealed that compared with that in the control group, the expression of *GFAP* in the DR group was significantly upregulated, and the expression of *GFAP* was effectively inhibited by ACT treatment (Fig 2D). Western blotting was used to detect the expression of GFAP and vimentin (which are involved in the construction of the cytoskeleton together with GFAP to maintain cell integrity and function) and Nestin (which may be reexpressed during reactive proliferation after injury, contributing to cell proliferation and differentiation) and revealed that, compared with those in the control group, the expression of GFAP, vimentin and Nestin in the DR group was upregulated, and the expression of these proteins was inhibited by ACT treatment (Fig 2E). Immunofluorescence detection of the colocalization of Kir4.1 and the Müller cell-specific marker GS revealed that the distribution of Kir4.1 was disrupted in the DR group, and the immunofluorescence staining was weak, whereas the fluorescence staining gradually increased after treatment with increasing concentrations of ACT (Fig 2F). In addition, the decrease in the expression of Kir4.1 in Müller cells during DR was an important cause of Müller cell swelling, and we found that Kir4.1 expression was significantly downregulated in the DR group compared with that in the control group and that Kir4.1 expression gradually increased after treatment with increasing ACT concentrations (Fig 2G and 2H). These findings illustrate that ACT improves hypertrophy and gliogenesis in Müller cells during DR.

### 3.3 ACT inhibits Kir4.1 channel-mediated reactive hyperplasia in Müller cells

**Fig 3:**
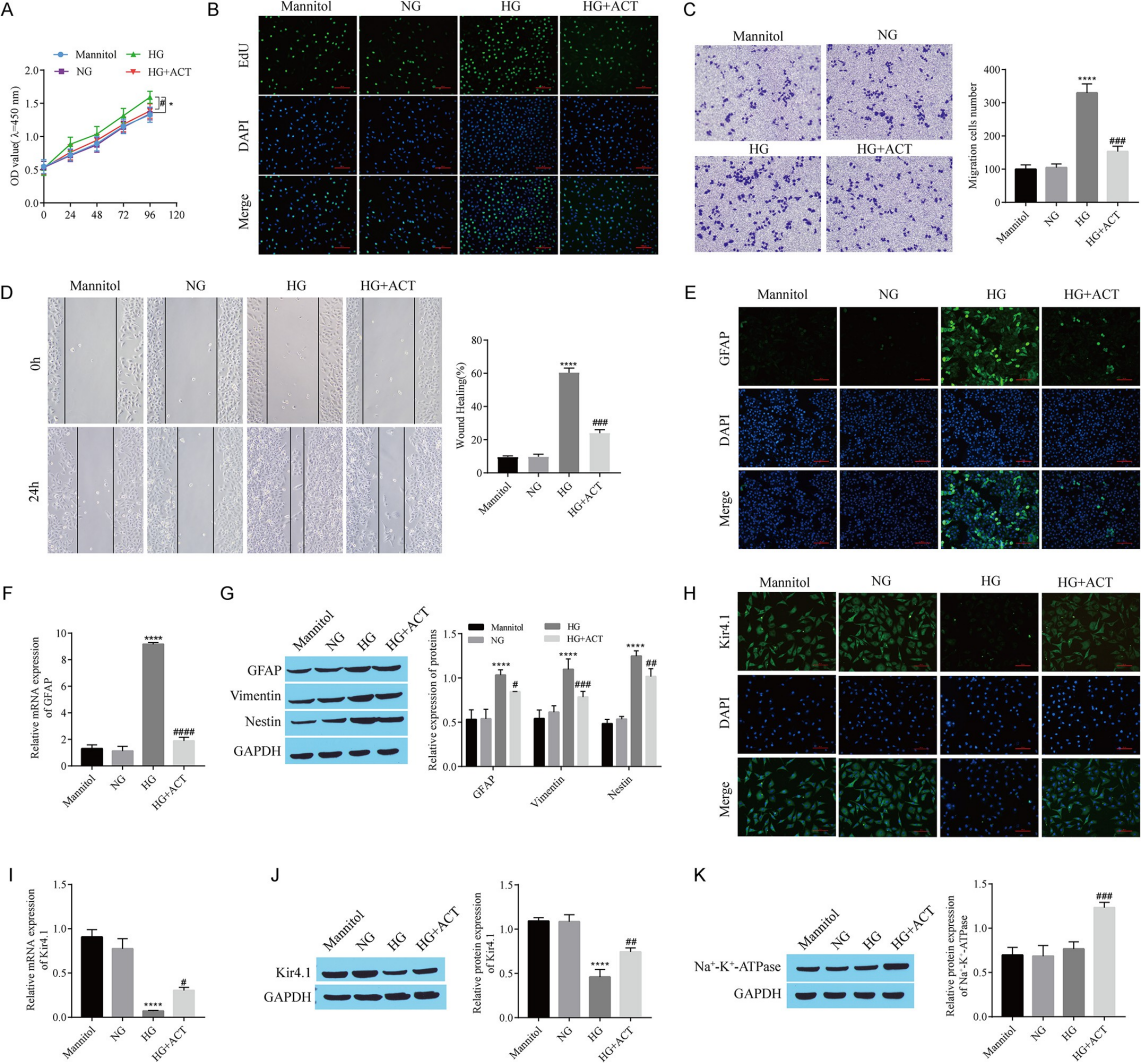
ACT inhibits Kir4.1 channel-mediated reactive hyperplasia in Müller cells. A: CCK-8 assay of cell proliferation. B: EdU staining for cell proliferation. C: Transwell assay of cell migration. D: Cell migration detected using cell scratch wound healing experiments. E: GFAP expression detected by immunofluorescence staining. F: RT‒qPCR analysis of *GFAP* expression. G: Western blots showing GFAP, Vimentin, and Nestin expression. H: Kir4.1 expression detected using immunofluorescence staining. I: RT‒qPCR analysis of *Kir4*.*1* expression. J: Kir4.1 expression detected by Western blotting. K: Western blot showing Na+-K+-ATPase expression. High glucose (33 mmol/L) treatment of RMC-1 cells was used to construct DR Cell model, and ACT (30 μM) treatment was used to observe its effects on RMC-1 proliferation, migration and other indicators.*P < 0.05 and ****P < 0.0001, compared with the NG group; ^#^P < 0.05, ^##^P < 0.01, ^###^P < 0.001, and ^####^P < 0.0001, compared with the HG group.

We further confirmed the effect of ACT on Kir4.1 channel-mediated reactive hyperplasia in Müller cells in a high glucose (HG)-induced DR cell model. CCK-8 and EdU assays revealed that the proliferation of Müller cells was greater in the HG group than in the normal glucose (NG) group and that ACT treatment reversed the effect of HG (Fig 3A and 3B). Similarly, Transwell and cell scratch wound healing assays of cell migration also revealed that the ability of HG to promote Müller cell migration was reversed by ACT (Fig 3C and 3D). The expression of GFAP, a marker of reactive proliferation in Müller cells, was detected by immunofluorescence staining and RT‒qPCR. Compared with that in the NG group, GFAP expression in the HG group was upregulated, while GFAP expression in the HG+ACT group was downregulated (Fig 3E and 3F). Western blot analysis revealed that the levels of GFAP, vimentin, and Nestin were elevated in the HG group compared with those in the control group; ACT treatment reversed the effect of HG (Fig 3G). Immunofluorescence, RT‒qPCR and Western blot detection of Kir4.1 expression revealed that HG treatment inhibited Kir4.1 expression, whereas ACT weakened the inhibitory effect of HG on Kir4.1 expression (Fig 3H‒3J). Compared with that in the NG group, the expression of Na+/K+-ATPase in the HG group was not obviously different; however, compared with that in the HG group, the level of Na+/K+-ATPase in the HG+ACT group was elevated (Fig 3K). In the whole experiment, we established an osmolality control group treated with mannitol, and all the experimental results were not significantly different from the experimental results for the mannitol and NG groups, which excluded the influence of osmolality on the results of the experiment. In summary, ACT restrains the reactive proliferation of Müller cells mediated by Kir4.1 channels.

### 3.4 TXNIP enhances Müller cell reactive hyperplasia and Kir4.1 channel dysfunction

**Fig 4:**
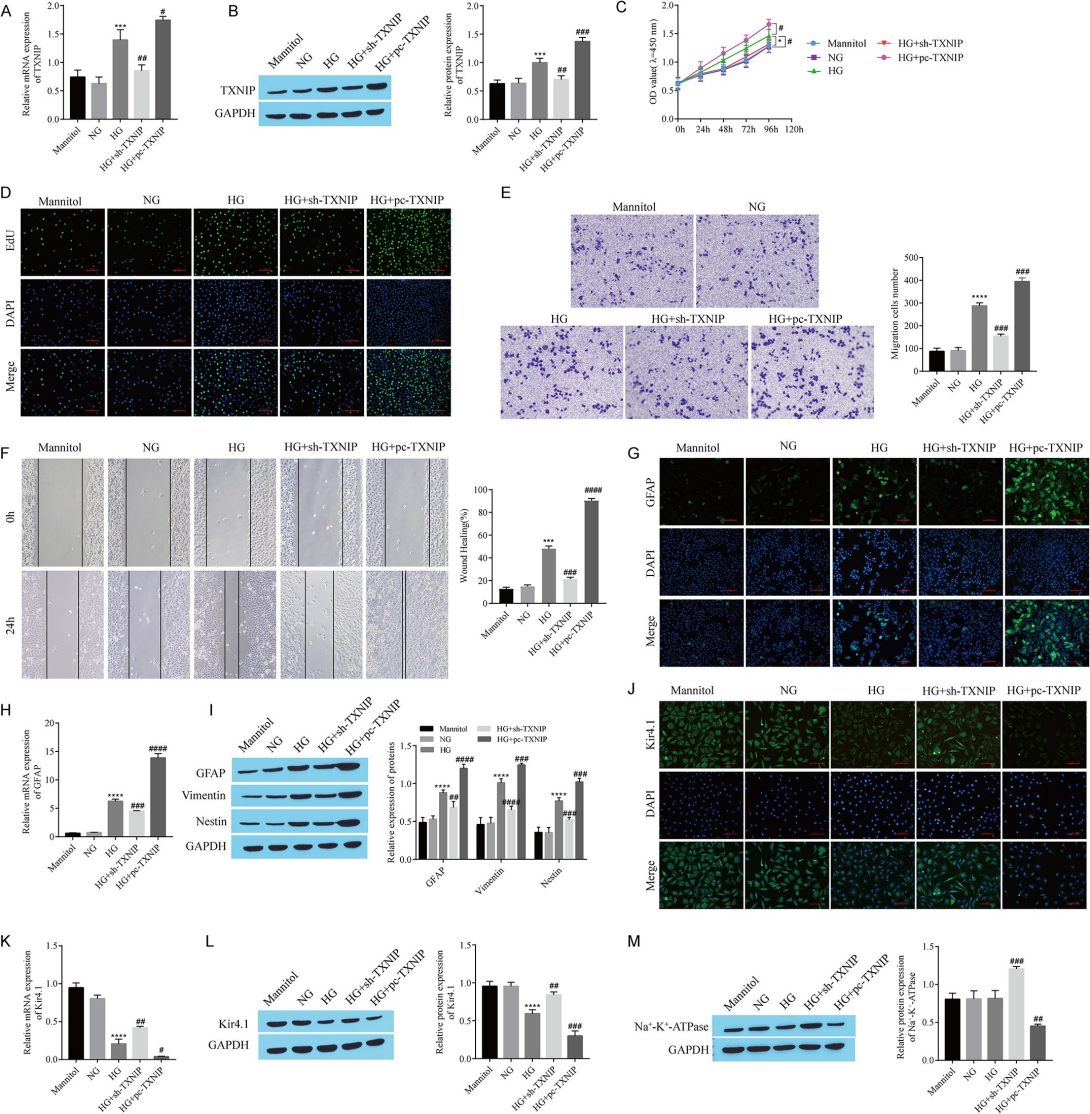
TXNIP enhances Müller cell reactive hyperplasia and Kir4.1 channel dysfunction. A: RT‒qPCR analysis of *TXNIP* expression. B: Western blot showing TXNIP expression. C: CCK-8 assay of cell proliferation. D: Cell proliferation detected by EdU staining. E: Transwell assay of cell migration. F: Cell scratch wound healing experiment to determine migration. G: GFAP expression detected by immunofluorescence staining. H: RT‒qPCR analysis of *GFAP* expression. I: Western blots showing the levels of GFAP, vimentin, and Nestin. J: Kir4.1 expression detected by immunofluorescence staining. K: RT‒qPCR analysis of *Kir4*.*1* expression. L: Western blot showing Kir4.1 expression. M: Western blot showing the level of Na^+^-K^+^-ATPase. RMC-1 cells were treated with high glucose (33 mmol/L) to construct DR cell models, and sh-TXNIP and pc-TXNIP were transfected into RMC-1 cells to observe their effects on the proliferation and migration of RMC-1 cells and the effect on Kir4.1 channels. *P < 0.05, ***P < 0.001, and ****P < 0.0001, compared with the NG group; ^#^P < 0.05, ^##^P < 0.01, ^###^P < 0.001, and ^####^P < 0.0001, compared with the HG group.

In the animal experiments, we noticed that the level of TXNIP was increased in the DR rats and that ACT treatment inhibited TXNIP expression. In cell experiments, we further explored the effect of TXNIP on reactive hyperplasia in Müller cells. Compared with that in the NG group, TXNIP expression was upregulated in the HG group, and TXNIP expression was significantly in the HG group after TXNIP knockdown and upregulated after TXNIP overexpression compared with that in the HG group (Fig 4A and 4B). CCK-8 and EdU assays were used to detect the proliferation of Müller cells, and TXNIP knockdown reversed the ability of HG to promote Müller cell proliferation, whereas TXNIP overexpression further enhanced the effect of HG (Fig 4C and 4D). Transwell and cell scratch wound healing assays also revealed that knocking down TXNIP reversed the effect of HG on promoting Müller cell migration, whereas overexpressing TXNIP had the opposite effect (Fig 4E and 4F). Immunofluorescence staining and RT‒qPCR revealed that TXNIP knockdown reversed the effect of HG on promoting GFAP expression, whereas TXNIP overexpression further enhanced the effect of HG on promoting GFAP expression (Fig 4G and 4H). The trends in the expression of GFAP, vimentin and Nestin detected using Western blotting were similar to those of GFAP detected using RT‒qPCR (Fig 4I). Similarly, TXNIP knockdown reversed the inhibitory effect of HG on Kir4.1 expression, whereas TXNIP overexpression had the opposite effect (Fig 4J–4L). Western blots indicated that, compared to the HG group, the level of Na^+^-K^+^-ATPase was increased in the HG+sh-TXNIP group and decreased in the HG+pc-TXNIP group (Fig 4M). These findings indicate that TXNIP enhances Müller cell reactive hyperplasia and Kir4.1 channel dysfunction.

### 3.5 ACT affects Müller cell reactive hyperplasia and Kir4.1 channel function by regulating TXNIP

**Fig 5:**
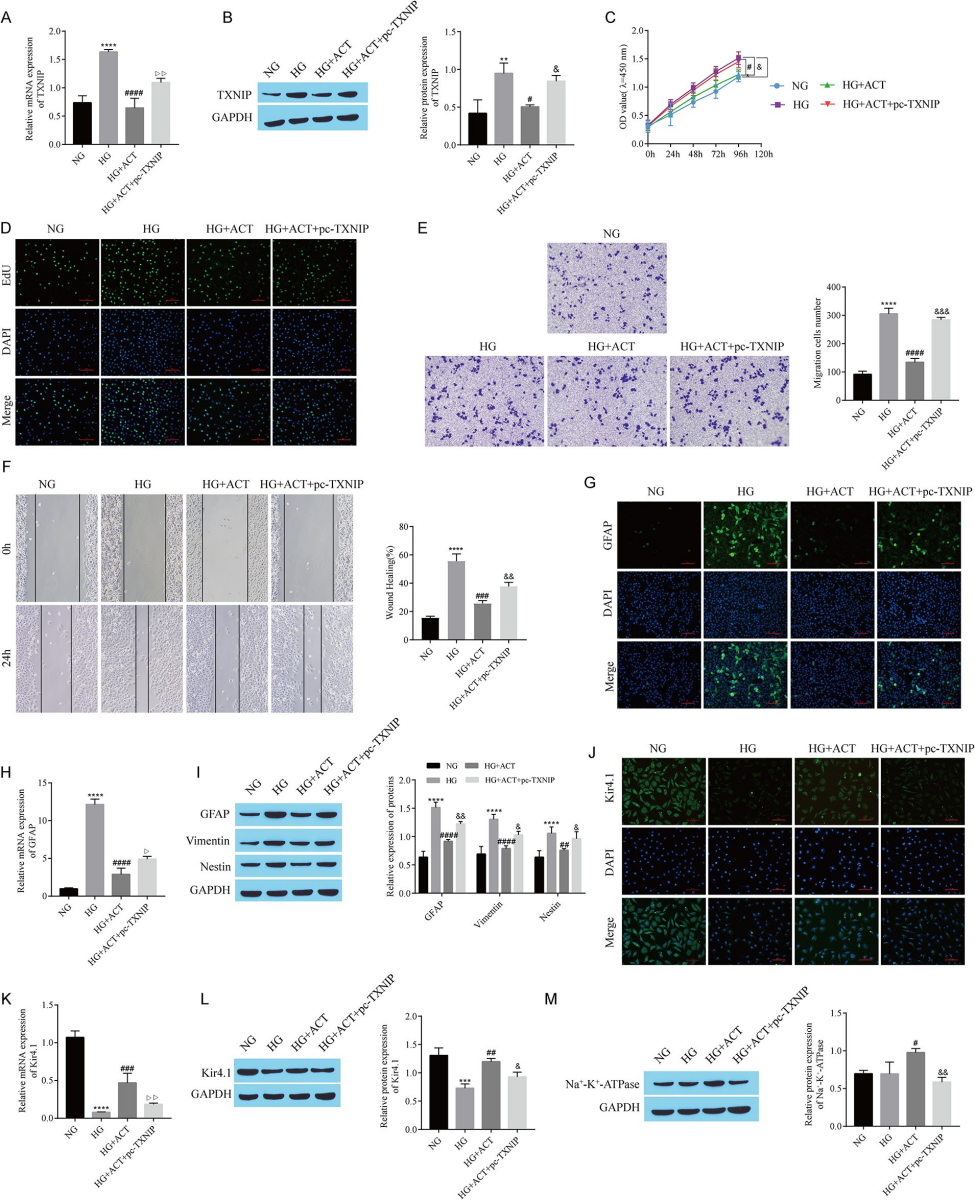
ACT affects Müller cell reactive hyperplasia and Kir4.1 channel function by regulating TXNIP. A: RT‒qPCR analysis of *TXNIP* expression. B: Western blot showing TXNIP expression. C: CCK-8 assay for cell proliferation. D: EdU staining for cell proliferation. E: Transwell assay for cell migration. F: Cell scratch wound healing experiment showing migration. G: GFAP expression detected by immunofluorescence staining. H: RT‒qPCR analysis of *GFAP* expression. I: Western blots showing the levels of GFAP, Vimentin, and Nestin. J: Kir4.1 expression detected by immunofluorescence staining. K: RT‒qPCR analysis of *Kir4*.*1* expression. L: Western blot showing Kir4.1 expression. M: Western blot showing the level of Na^+^-K^+^-ATPase. RMC-1 cells were treated with high glucose (33 mmol/L) to construct a DR Cell model, RMC-1 cells were treated with ACT (30 μM), and then transfected with pc-TXNIP to observe the effect of ACT. *P < 0.05, ***P < 0.001, and ****P < 0.0001, compared with the NG group; ^#^P < 0.05, ^##^P < 0.01, ^###^P < 0.001, and ^####^P < 0.0001, compared with the HG group; ^&^P < 0.05, ^&&^P < 0.01, and ^&&&^P < 0.001, compared with the HG+ACT group.

ACT was further confirmed to affect reactive hyperplasia in Müller cells and the function of Kir4.1 channels via the modulation of TXNIP. RT‒qPCR and Western blotting revealed that the increase in TXNIP expression induced by HG could be reversed by ACT, whereas the overexpression of TXNIP reversed the effect of ACT (Fig 5A and 5B). CCK-8 and EdU assays revealed that TXNIP overexpression effectively reversed the inhibitory effect of ACT on Müller cell proliferation (Fig 5C and 5D). The detection of Müller cell migration also revealed decreased migration of cells in the HG+ACT group compared to the HG group, and the number of migrating cells in the HG+ACT+pc-TXNIP group was greater than that in the HG+ACT group (Fig 5E and 5F). GFAP expression was downregulated in the HG+ACT group and upregulated in the HG+ACT+pc-TXNIP group compared with the HG+ACT group (Fig 5G and 5H). The trend in the protein expression of GFAP, vimentin, and Nestin, as determined by Western blotting, was the same as that in the protein expression of GFAP, as detected using RT‒qPCR (Fig 5I). Immunofluorescence staining, RT‒qPCR and western blotting revealed that Kir4.1 expression was upregulated in the HG + ACT group compared with the HG group, and the level of Kir4.1 in the HG + ACT + pc-TXNIP group was downregulated compared with that in the HG + ACT group (Fig 5J‒5L). The expression of Na^+^-K^+^-ATPase determined by western blotting was similar to that of Kir4.1 (Fig 5M). These results illustrate that ACT affects reactive hyperplasia in Müller cells and the function of Kir4.1 channels through the regulation of TXNIP.

### 3.6 TXNIP promotes Müller cell reactive hyperplasia and reduces Kir4.1 channel function by restraining the PI3K/AKT pathway

**Fig 6:**
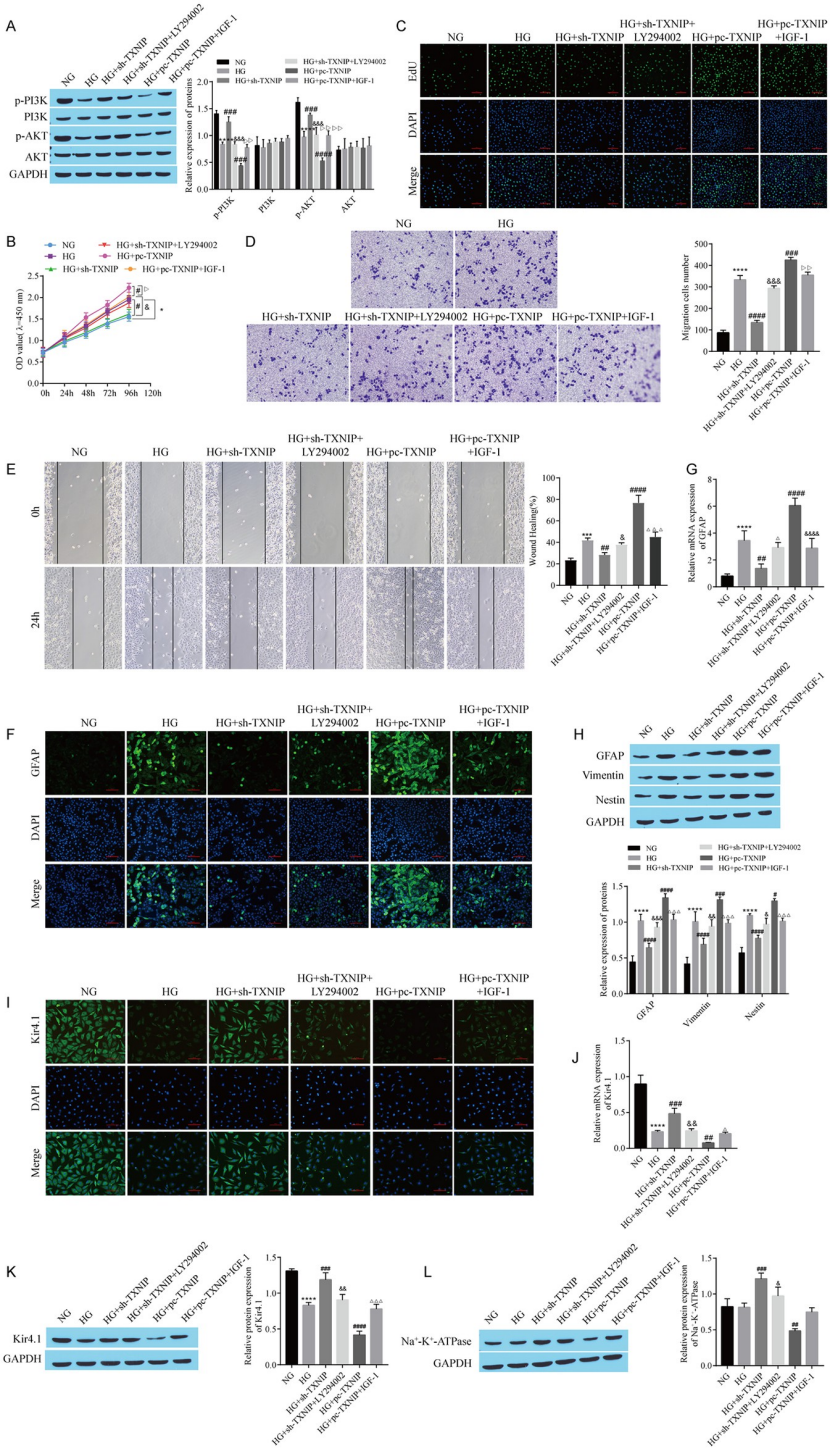
TXNIP promotes Müller cell reactive hyperplasia and reduces Kir4.1 channel function by restraining the PI3K/AKT pathway. A: Western blots showing the levels of p-PI3K, PI3K, p-AKT, and AKT. B: CCK-8 assay for cell proliferation. C: EdU staining for cell proliferation. D: Transwell assay of cell migration. E: Cell scratch wound healing experiment for migration. F: GFAP expression was detected by immunofluorescence staining. G: RT‒qPCR analysis of *GFAP* expression. H: Western blots showing the levels of GFAP, Vimentin, and Nestin. I: Kir4.1 expression was detected by immunofluorescence staining. J: RT‒qPCR for testing *Kir4*.*1* levels. K: Western blot showing Kir4.1 levels. L: Western blot showing the expression of Na^+^-K^+^-ATPase. RMC-1 cells were treated with high glucose (33 mmol/L) to construct DR cell models, and RMC-1 cells were transfected with sh-TXNIP and pc-TXNIP, and RMC-1 cells were treated with PI3K/Akt inhibitor LY294002 (10 μM) and PI3K/Akt inhibitor activator IGF-1, respectively. *P < 0.05, **P < 0.01, ***P < 0.001, and ****P < 0.0001, compared with the NG group; ^#^P < 0.05, ^##^P < 0.01, ^###^P < 0.001, and ^####^P < 0.0001, compared with the HG group; ^&^P <0.05, ^&&^P <0.01, and ^&&&^P <0.001, compared with the HG+sh-TXNIP group; ^Δ^P <0.05, ^ΔΔ^P <0.01, ^ΔΔΔ^P <0.001, and ^ΔΔΔΔ^P<0.0001, compared with the HG+ pc-TXNIP group.

The expression of Kir4.1 is associated with the N-methyl-D-aspartate receptor (NMDAR), which is regulated by the PI3K/Akt pathway; therefore, we further determined whether TXNIP affects Müller cell reactive proliferation and Kir4.1 channel function through the PI3K/AKT pathway. Western blot detection of the levels of proteins in the PI3K/AKT pathway showed that compared with NG group, the levels of p-PI3K and p-Akt in the HG group were decreased, and TXNIP knockdown effectively restored the expression of p-PI3K and p-Akt. TXNIP overexpression further decreased the levels of p-PI3K and p-Akt. In addition, treatment with the PI3K/AKT pathway inhibitor LY294002 effectively reversed the effect of TXNIP knockdown, and treatment with the PI3K/AKT pathway activator IGF-1 also reversed the effect of TXNIP overexpression. Moreover, no significant difference in the expression of PI3K or AKT was observed among the groups (Fig 6A). CCK-8 and EdU assays showed that compared with the HG group, the proliferation of cells in the HG+sh-TXNIP group decreased, and the proliferation of cells in the HG+pc-TXNIP group was further increased, whereas LY294002 and IGF-1 treatments reversed the effects of TXNIP knockdown and overexpression, respectively (Fig 6B and 6C). Transwell and cell scratch wound healing assays revealed that cell migration was decreased in the HG+sh-TXNIP group and further increased in the HG+pc-TXNIP group compared with the HG group, whereas LY294002 and IGF-1 treatments reversed the effects of TXNIP knockdown and overexpression, respectively (Fig 6D and 6E). The detection of GFAP expression revealed that the induction of GFAP expression by HG was weakened by sh-TXNIP and enhanced by pc-TXNIP, the effect of sh-TXNIP was reversed by LY294002, and the effect of pc-TXNIP was reversed by IGF-1 (Fig 6F and 6G). The alterations in the levels of the GFAP, Vimentin, and Nestin proteins detected via western blotting coincided with the changes in GFAP expression detected via RT‒qPCR (Fig 6H). Compared with that in the HG group, the expression of Kir4.1 in the HG+sh-TXNIP group was upregulated, the expression of Kir4.1 in the HG+pc-TXNIP group was downregulated, LY294002 treatment reversed the effect of sh-TXNIP, and IGF-1 treatment reversed the effect of pc-TXNIP (Fig 6I–6K). The alteration in the level of Na^+^-K^+^-ATPase detected by Western blotting was coincident with the trend observed for Kir4.1 (Fig 6L). These findings suggest that TXNIP facilitates the reactive proliferation of Müller cells and reduces the function of Kir4.1 channels by restraining the PI3K/AKT pathway.

### 3.7 TXNIP promotes the reactive proliferation of retinal Müller cells and Kir4.1 channel impairment in vivo

**Fig 7:**
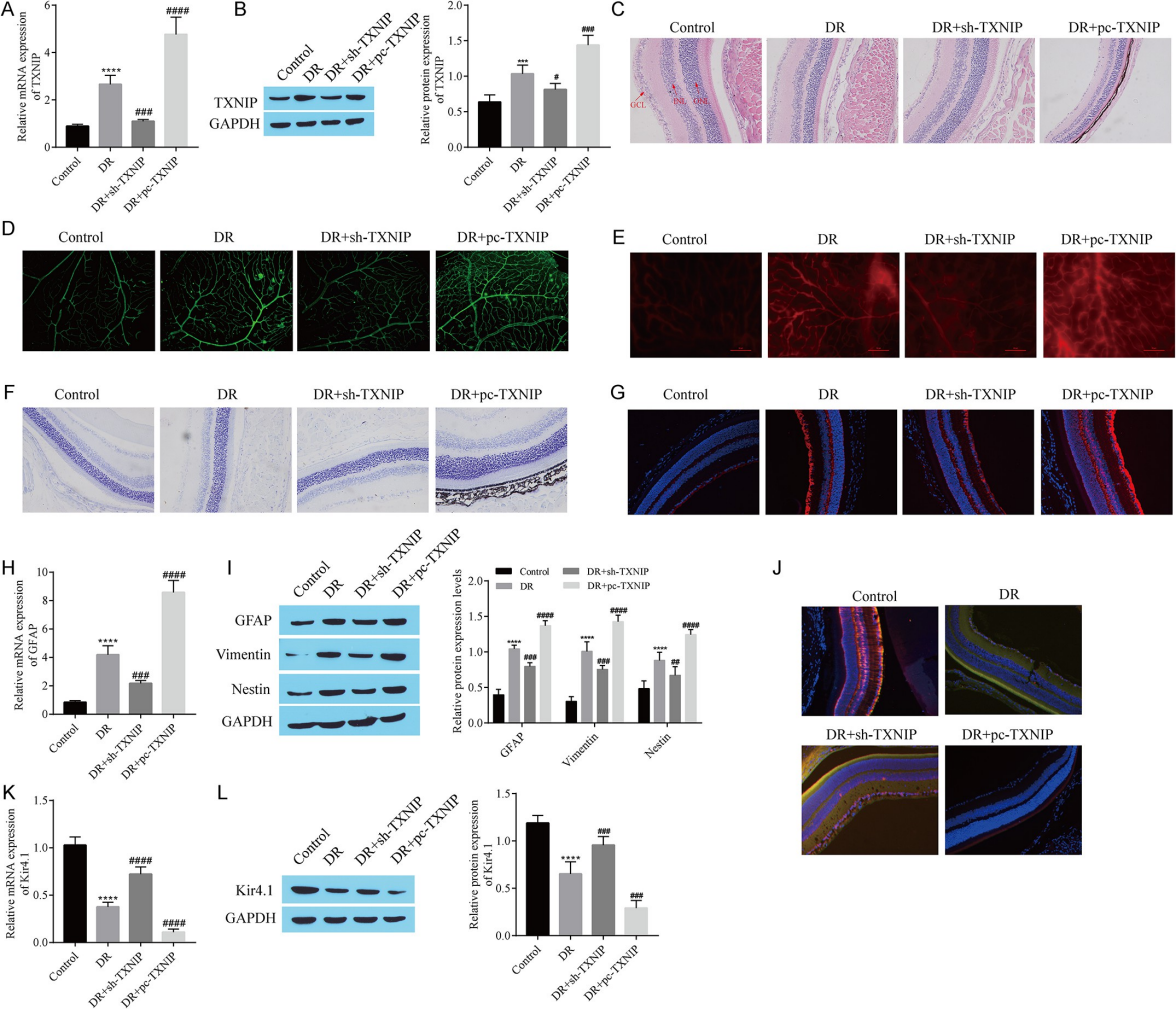
TXNIP promotes the reactive proliferation of retinal Müller cells and Kir4.1 channel impairment in vivo. A: RT‒qPCR detection of *TXNIP* expression. B: Western blot showing TXNIP expression. C: Retinal morphological and structural changes observed via HE staining. D: The morphology of retinal blood vessels was observed by retinal smearing and fluorescent staining. E: Evans blue staining of the vascular permeability of the retina. F: Müller cells observed in semithin sections of the retina. G: GFAP expression detected via immunofluorescence staining. H: *GFAP* expression detected via RT‒qPCR. I: Western blots showing the levels of GFAP, Vimentin, and Nestin. J: Double immunofluorescence staining showing the levels of Kir4.1 and GS. K: *Kir4*.*1* levels detected using RT‒qPCR. L: Western blot showing Kir4.1 expression. DR Rats were constructed by peritoneal injection of STZ (60 mg/kg), and adenovirus containing sh-TXNIP and pc-TXNIP plasmids were injected into the tail vein to observe the effects on DR Process and detect various indicators. ***P < 0.001, and ****P < 0.0001, compared with the control group; ^#^P < 0.05, ^##^P < 0.01, ^###^P < 0.001, and ^####^P < 0.0001, compared with the DR group.

Animal experiments further confirmed the effects of TXNIP on Müller cell reactive hyperplasia and Kir4.1 channels. RT‒qPCR and western blotting revealed that, compared with that in the DR group, TXNIP expression was lower in the DR+sh-TXNIP group and higher in the DR+pc-TXNIP group (Fig 7A and 7B). Changes in the retinal morphological structure were observed via HE staining. Compared with the control group, the retinal structure of the DR group was damaged, the stratification was not clear, and the ganglion cell arrangement was disordered. Compared with the DR group, the DR+sh-TXNIP group exhibited alleviated retinal damage, and the retinal damage in the DR+pc-TXNIP group was more severe (Fig 7C). Observations of vascular morphology revealed clustered microhemangiomas around retinal vessels in the DR group, whereas in the DR+sh-TXNIP group, the vascular wall was relatively smooth, the number of clustered microhemangiomas decreased, and the number of clustered microhemangiomas in the DR+pc-TXNIP group was further increased (Fig 7D). In addition, the permeability of blood vessels in the DR group was obvious; however, compared with that in the DR group, the permeability of blood vessels in the DR+sh-TXNIP group was decreased, and the permeability of blood vessels in the DR+pc-TXNIP group was further increased (Fig 7E). Müller cell hypertrophy and edema were observed in semithin sections of the retina. The results revealed that fluid accumulation in the form of a band-like clear gap was detected between the nuclei of the outer nuclear layer in the DR group; compared with that in the DR group, the band-like clear gap was less intense in the DR+sh-TXNIP group but was greater in the DR+pc-TXNIP group (Fig 7F). Immunofluorescence staining and RT-qPCR were used to detect the expression of GFAP, and GFAP expression was increased in the DR group but decreased in the DR+sh-TXNIP group, and the expression of GFAP in the DR+pc-TXNIP group was further increased compared with that in the DR group (Fig 7G and 7H). The trend in GFAP, vimentin, and Nestin expression detected using Western blotting was consistent with the trend for GFAP expression detected using RT‒qPCR (Fig 7I). Immunofluorescence detection of the colocalization of Kir4.1 and the Müller cell-specific marker GS revealed that, in the DR group, the distribution of Kir4.1 was disrupted, and the immunofluorescence staining was weak. Compared with the DR group, the fluorescence staining in the TXNIP group was restored to some extent, while the fluorescence staining in the DR+pc-TXNIP group was weaker (Fig 7J). Detection of Kir4.1 expression via RT‒qPCR and western blotting revealed that the Kir4.1 level was lower in the DR group than in the control group, was upregulated in the DR+sh-TXNIP group compared with the DR group, and was downregulated in the DR+pc-TXNIP group (Fig 7K and 7L). These findings indicate that TXNIP facilitates the reactive proliferation of retinal Müller cells and Kir4.1 channel dysfunction in vivo.

### 3.8 ACT alleviates retinal dysfunction in a diabetes model by inhibiting TXNIP-mediated activation of the PI3K/AKT pathway

**Fig 8:**
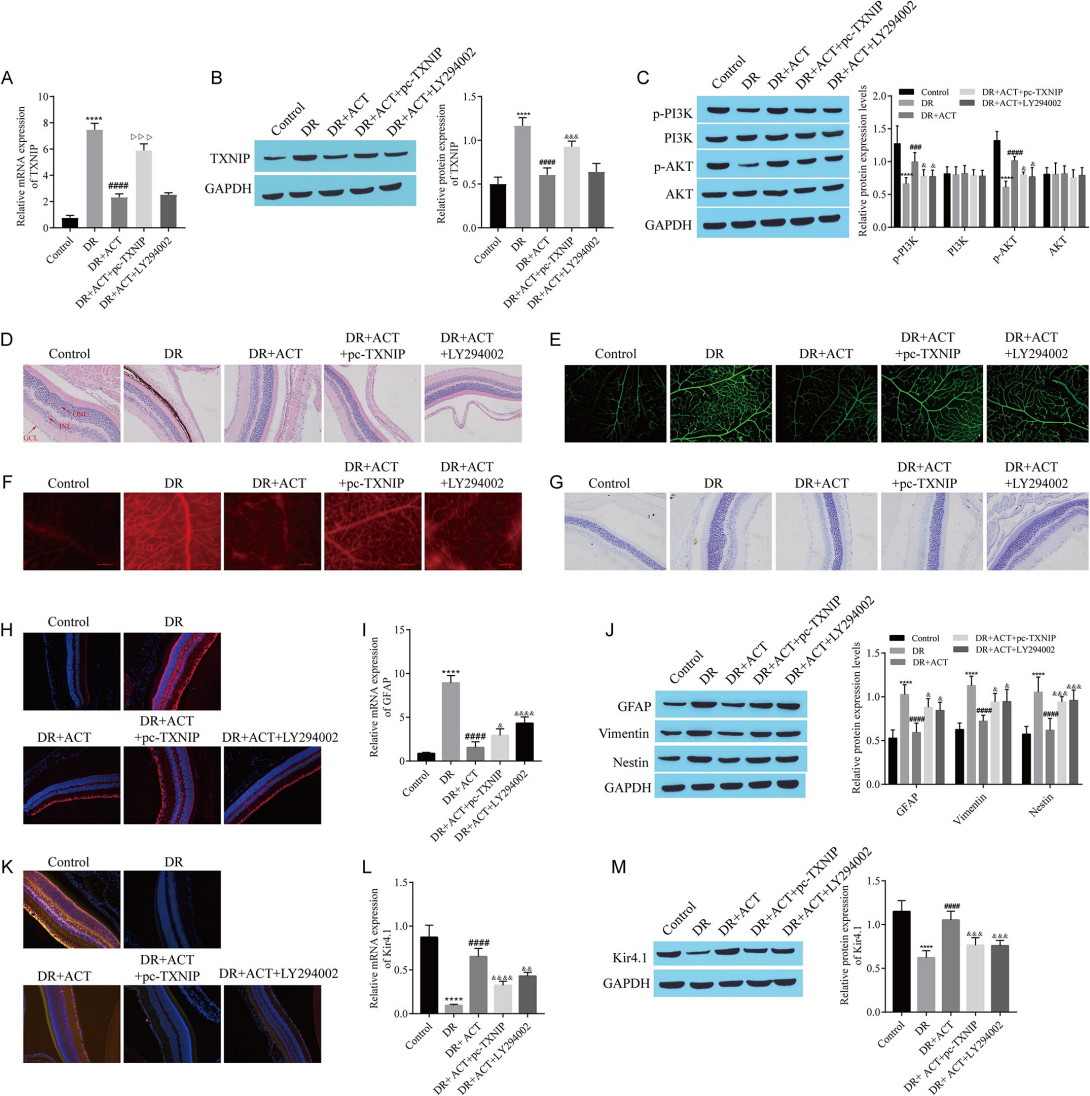
ACT alleviates retinal dysfunction in a diabetes model by inhibiting TXNIP-mediated activation of the PI3K/AKT pathway. A: RT‒qPCR for *TXNIP* expression. B: Western blot showing TXNIP expression. C: Western blots showing the levels of p-PI3K, PI3K, p-AKT, and AKT. D: Changes in retinal morphological structure observed with HE staining. E: The morphology of retinal blood vessels was observed by retinal smearing and fluorescent staining. F: Evans blue staining to detect the permeability of retinal blood vessels. G: Müller cells observed in semithin sections of the retina. H: GFAP expression detected via immunofluorescence staining. I: RT‒qPCR for the detection of *GFAP* expression. J: Western blots showing the levels of GFAP, Vimentin, and Nestin. K: The expression of Kir4.1 and GS observed via double immunofluorescence staining. L: Detection of *Kir4*.*1* via RT‒qPCR. M: Western blot showing Kir4.1 expression. DR Rats were constructed by peritoneal injection of STZ (60 mg/kg). After ACT treatment, adenovirus containing pc-TXNIP plasmid and PI3K/Akt inhibitor LY294002 were injected into the tail vein to observe the effect of the treatment on ACT. ****P < 0.0001, compared with the control group; ^###^P < 0.001 and ^####^P < 0.0001, compared with the DR group; ^&^P < 0.05, ^&&^P < 0.01, and ^&&^P < 0.001, compared with the DR+ACT group.

We subsequently investigated the relationships between ACT treatment and TXNIP and the PI3K/AKT pathway in vivo. RT‒qPCR and Western blotting revealed that ACT inhibited the upregulation of TXNIP, whereas pc-TXNIP reversed this effect; however, LY294002, an inhibitor of the PI3K/AKT pathway, did not affect the inhibitory effect of ACT on TXNIP expression (Fig 8A and 8B). Western blot analysis revealed that ACT restored the expression of p-PI3K and p-AKT, but pc-TXNIP and LY294002 inhibited the effect of ACT, while the expression of PI3K and AKT did not change significantly between the groups (Fig 8C). HE staining and the retinal vascular analysis revealed that ACT improved retinopathy, but pc-TXNIP and LY294002 reversed these improvements (Fig 8D–8F). Semithin retinal sections revealed that ACT reduced edema in Müller cells, which was likewise reversed by pc-TXNIP and LY294002 (Fig 8G). Immunofluorescence staining and RT‒qPCR of GFAP revealed that ACT inhibited GFAP expression, but pc-TXNIP and LY294002 restored GFAP expression (Fig 8H and 8I). The trends in GFAP, vimentin, and Nestin expression detected using Western blots were consistent with the trend in GFAP expression detected using RT‒qPCR (Fig 8J). Immunofluorescence staining revealed that ACT enhanced the colocalization of Kir4.1 and GS, but pc-TXNIP and LY294002 attenuated this change (Fig 8K). RT‒qPCR and Western blotting revealed that ACT upregulated the expression of Kir4.1, whereas pc-TXNIP and LY294002 downregulated Kir4.1 expression (Fig 8L and 8M). In summary, ACT alleviates diabetic retinal dysfunction by inhibiting TXNIP and activating the PI3K/AKT pathway.

## 4 Discussion

DR is not only a microvascular illness but also a BRB disease, and Müller cell-derived VEGF is a master regulator of BRB function [26]. In addition, Müller cells run longitudinally through the entire neural retina, and their specialized foot plates can adhere to the retinal capillary wall to participate in the formation of the BRB [27]. Therefore, based on the important role of Müller cells in DR pathology, we investigated the molecular mechanism of ACT in DR from the perspective of the reactive hyperplasia of Müller cells.

ACT, a natural compound, has been confirmed to have anticancer activity and is efficacious in the treatment of various cancers by regulating various pathways [28]. Our previous studies revealed that ACT relieves glaucoma and traumatic optic neuropathy by attenuating retinal ganglion cell (RGC) damage [29]. In this study, we reported that ACT effectively relieved DR in rats and reduced the formation of retinal hemangiomas and the permeability of retinal blood vessels in a concentration-dependent manner. Furthermore, we also noticed that ACT improved the degree of Müller cell hypertrophy and glial production. The therapeutic effect of ACT on DR observed in this study was consistent with that reported by Yang et al. [9]. However, by analyzing Müller cell reactive hyperplasia, we elucidated a novel molecular mechanism of action of ACT in the treatment of DR.

Reactive hyperplasia of Müller cells is a nonspecific response to retinal injury caused by Müller cell activation [30]. Moreover, reactive hyperplasia of Müller cells is an important pathological lesion in DR development. Studies have shown that inhibiting Müller cell proliferation can effectively ameliorate retinal damage. For example, Jing Xie et al. reported that olfactory ensheathing cells (OECs) improve the visual function of royal college of surgeon (RCS) rats by mediating the Notch pathway to restrain the proliferation of Müller cells and gliosis [31]. Therefore, Müller cells could be latent cellular targets for DR treatment. GFAP overexpression is a sensitive marker of Müller cell reactive hyperplasia [32]. Eunsoo Jung et al. noted that aloin, which prevents Müller cell gliosis, could decrease the level of GFAP in Müller cells in a dose-dependent manner [33]. Other studies have shown that increased expression of intermediate filaments (mainly GFAP, Vimentin, and Nestin) and glial cell hypertrophy are the most prominent features of reactive gliosis [34]. This study found that ACT treatment effectively decreased the levels of GFAP, Vimentin and Nestin in a dose-dependent manner. These findings illustrate that ACT improves Müller cell hypertrophy and gliogenesis during DR progression. In the condition of retinal injury, Müller cells will not only undergo reactive hyperplasia, but also down-regulation of Kir channels and functional inactivation [35]. Kir4.1 is a member of the Kir4.x family and is mainly responsible for the transport of potassium ions among Kir channels [36]. Kir4.1 is distributed mainly on Müller cells in the retina. When the function of Kir4.1 is impaired, the inability to secrete excess potassium ions causes hyperosmotic pressure to increase water influx and cause the swelling of Müller cells [37]. Previous studies have shown that the retinas of diabetic animals exhibit reduced expression of Kir4.1 around blood vessels [38]. This study revealed that in DR rat retinal tissues, the distribution of Kir4.1 was disrupted, and its expression was prominently reduced; however, ACT treatment restored Kir4.1 expression. We also observed that Na^+^/K^+^-ATPase expression in Müller cells was unchanged after HG treatment, possibly due to the decreased expression of Kir4.1 under HG conditions. After ACT treatment, the upregulation of Kir4.1 may in turn promote the upregulation of Na^+^/K^+^-ATPase expression.

TXNIP is upregulated in HG-treated Müller cells and mediates oxidative stress and inflammation in Müller cells [39]. This study revealed that TXNIP expression was upregulated in DR rat retinal tissues and HG-treated Müller cells and that the overexpression of TXNIP could enhance Müller cell reactive hyperplasia and Kir4.1 channel dysfunction. Studies have also shown that TXNIP actively regulates autophagy by restraining the PI3K/AKT/mTOR pathway [22]. Kir4.1 may be activated in a PI3K-dependent manner [18]. In addition, Kir4.1 expression is interrelated with the modulation of PI3K/Akt pathway through NMDARs [40]. This study revealed that the overexpression of TXNIP effectively restrained the expression of p-PI3K and p-Akt and that the effects of TXNIP overexpression on Müller cell reactive hyperplasia and Kir4.1 channel dysfunction could be reversed by the PI3K/Akt activator IGF-1. Studies have shown that ACT can increase the activity of the PI3K/AKT pathway in multiple cell and animal models [41, 42]. This study revealed that ACT decreased TXNIP expression, and the activation of the PI3K/AKT pathway and the alleviation of retinal dysfunction in DR rats were reversed by the overexpression of TXNIP. Therefore, ACT activates the PI3K/AKT pathway via the suppression of TXNIP to restrain Müller cell reactive hyperplasia and block the progression of DR.

In summary, this study revealed that ACT can effectively inhibit Müller cell reactive proliferation and alleviate retinal dysfunction in DR rats and that the specific therapeutic molecular mechanism involves ACT inhibiting Müller cell reactive hyperplasia by regulating TXNIP and mediating the expression of the Kir4.1 channel in a PI3K/Akt-dependent manner to alleviate diabetic retinopathy.

## Supporting information

S1 Raw image (PDF)
