# Supplementary material for: Acteoside relieves diabetic retinopathy through the inhibition of Müller cell reactive hyperplasia by regulating TXNIP and mediating Kir4.1 channels in a PI3K/Akt-dependent manner
Source: PLoS One. 2024 Dec 17;19(12):e0312565. doi: 10.1371/journal.pone.0312565 (PMC11651567; doi:10.1371/journal.pone.0312565)

Figure 1G

TXNIP (44 kDa)

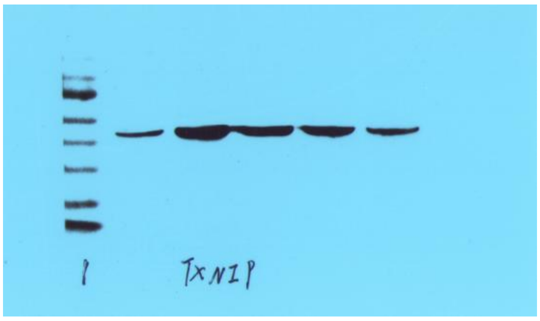

GAPDH (37 kDa)

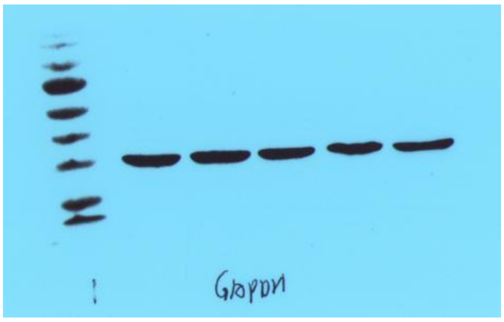

Figure 1H

p-PI3K (126 kDa)

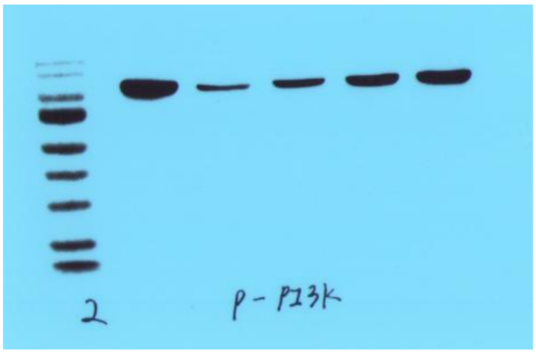

PI3K (126 kDa)

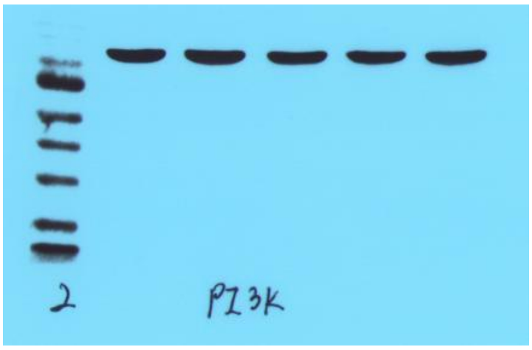

p-AKT (56 kDa)

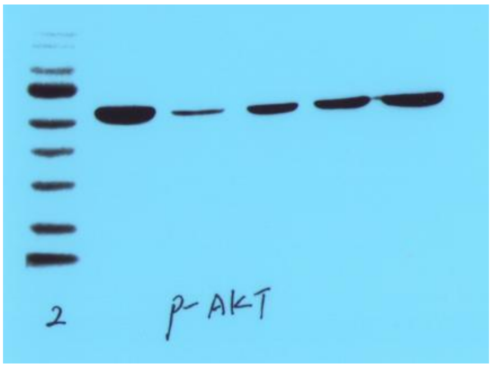

AKT (56 kDa)

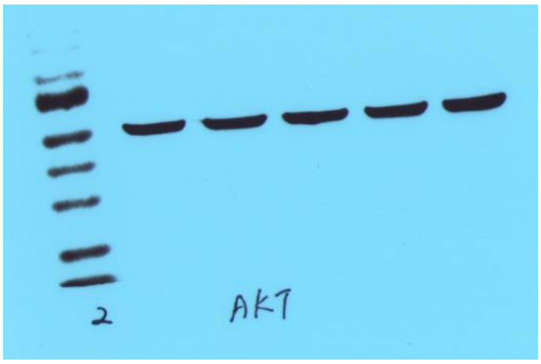

GAPDH (37 kDa)

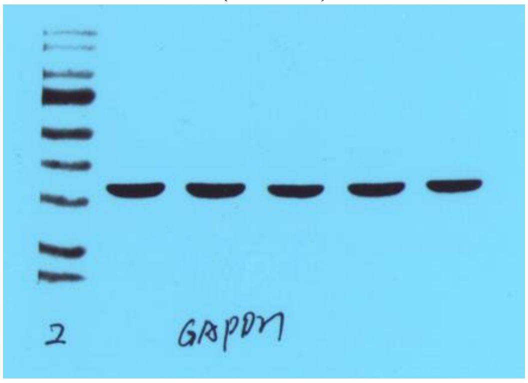

Figure 2E

GFAP (50 kDa)

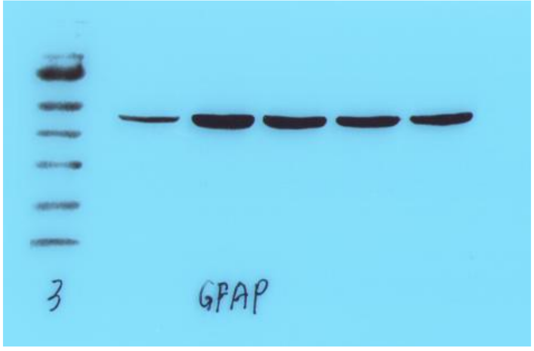

Vimentin (54 kDa)

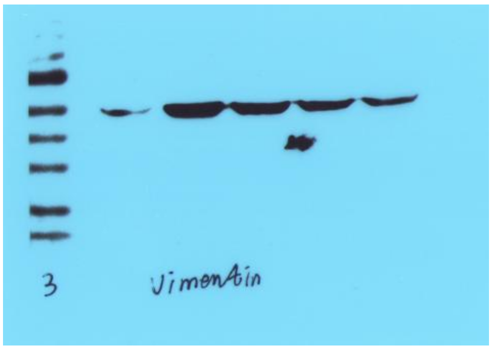

Nestin (177 kDa)

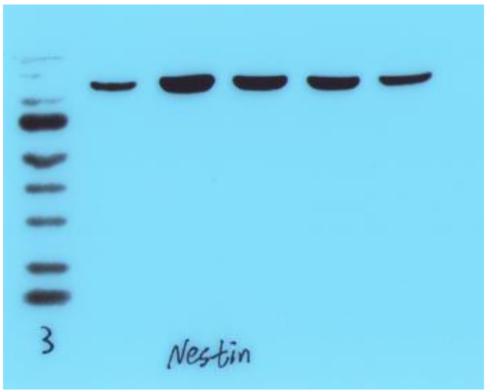

GAPDH (37 kDa)

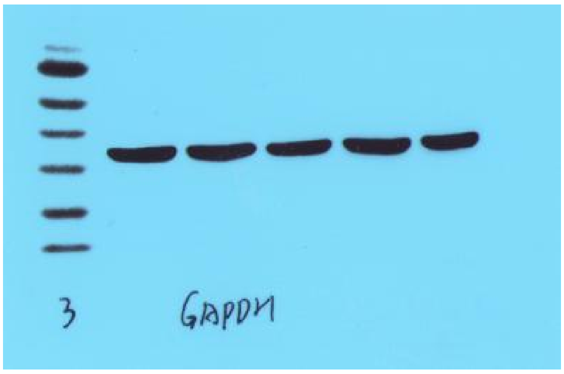

Figure 2H

Kir4.1 (42 kDa)

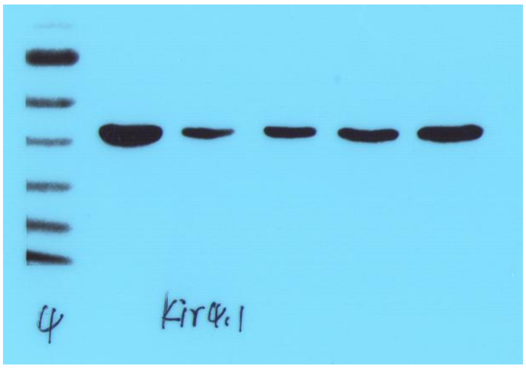

GAPDH (37 kDa)

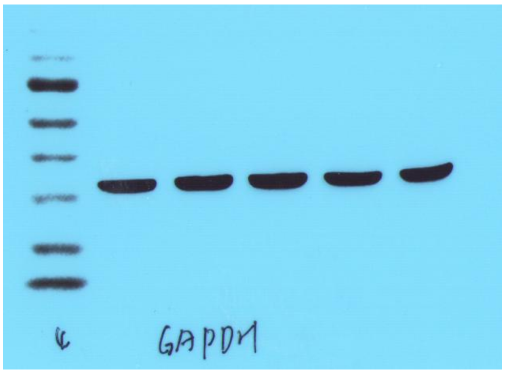

Figure 3G

GFAP (50 kDa)

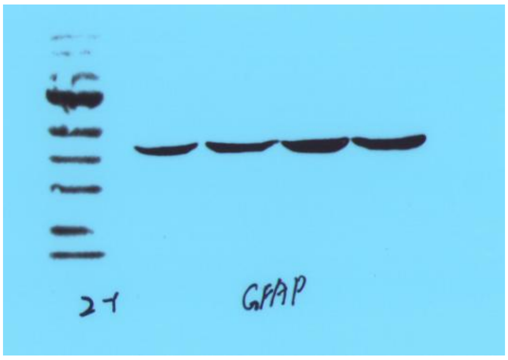

Vimentin (54 kDa)

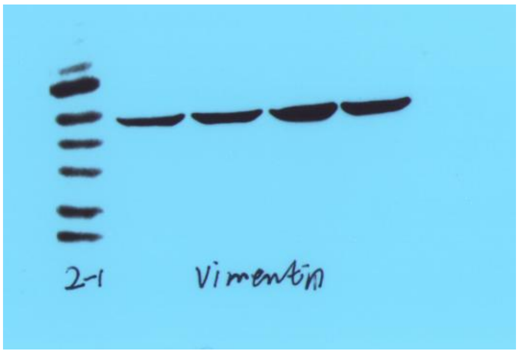

Nestin (177 kDa)

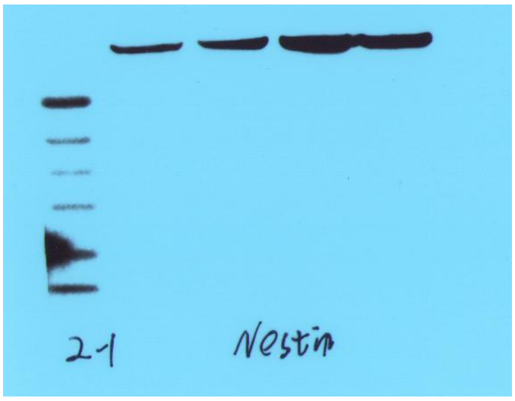

GAPDH (37 kDa)

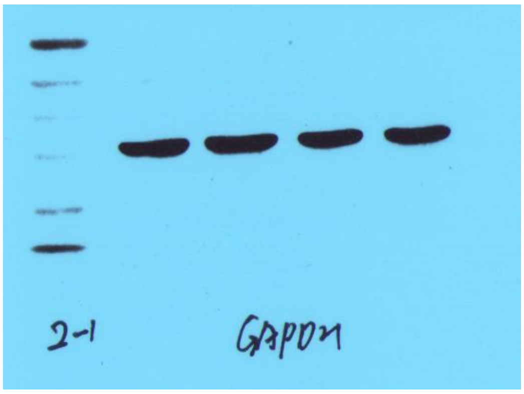

Figure 3J

Kir4.1 (42 kDa)

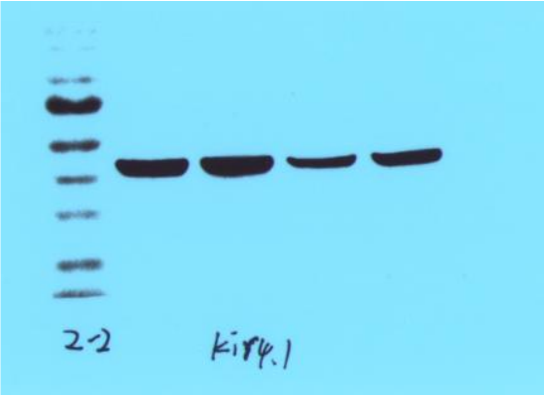

GAPDH (37 kDa)

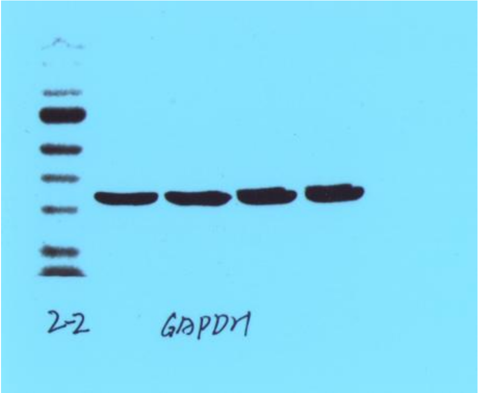

Figure 3K

Na^+-K^+-ATPase (30 kDa)

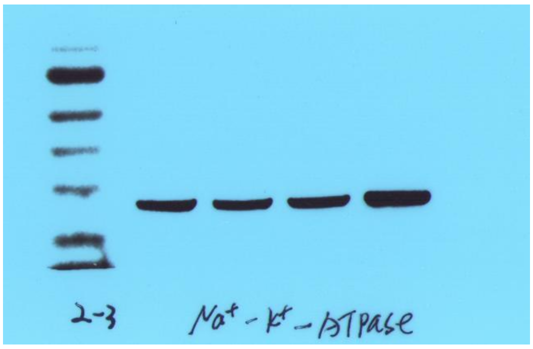

GAPDH (37 kDa)

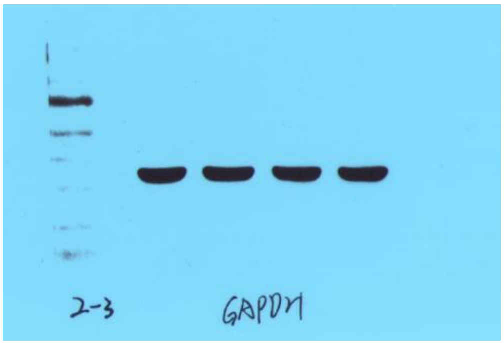

Figure 4B

TXNIP (44 kDa)

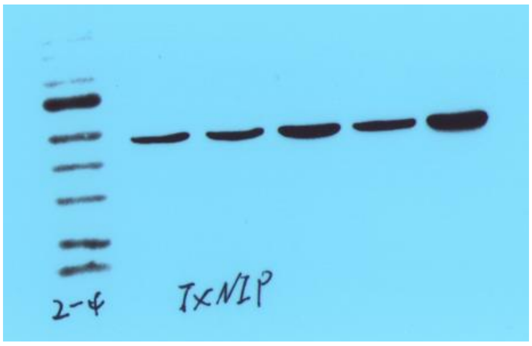

GAPDH (37 kDa)

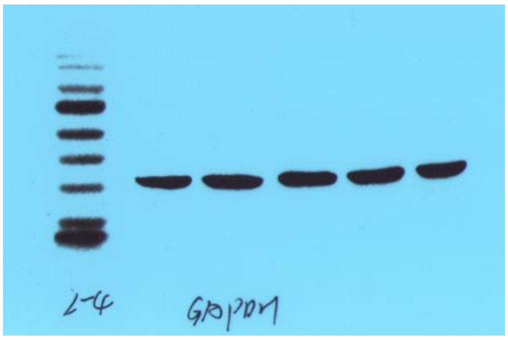

Figure 4I

GFAP (50 kDa)

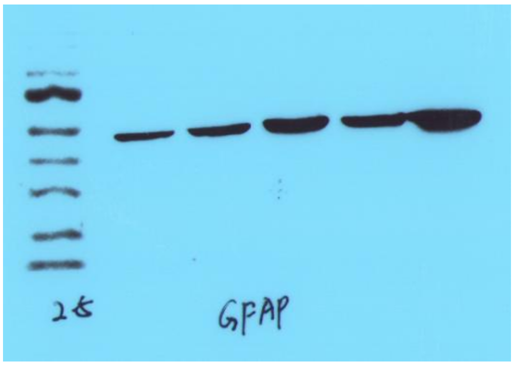

Vimentin (54 kDa)

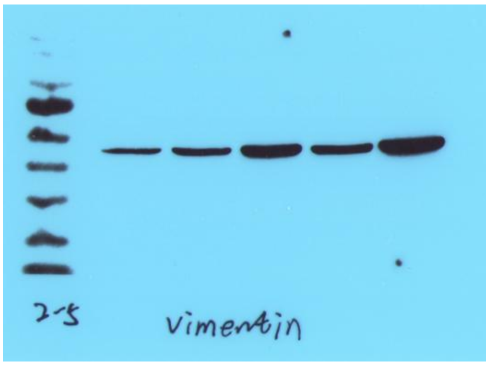

Nestin (177 kDa)

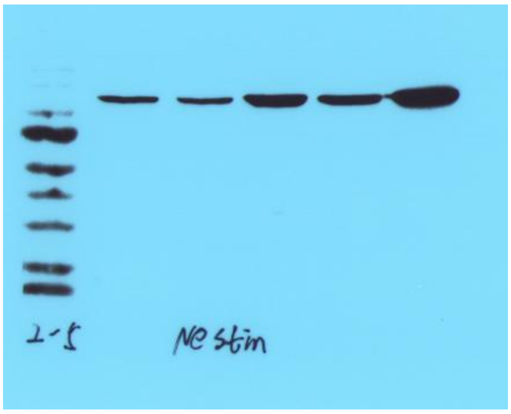

GAPDH (37 kDa)

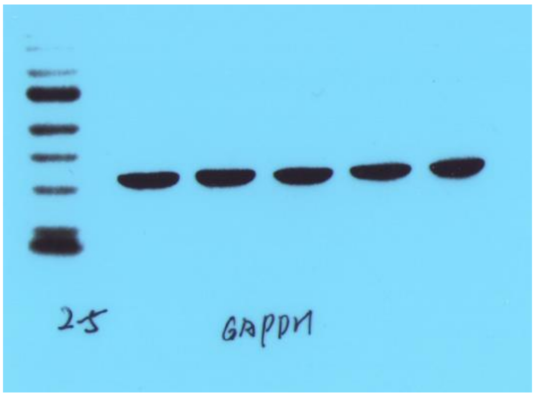

Figure 4L

Kir4.1 (42 kDa)

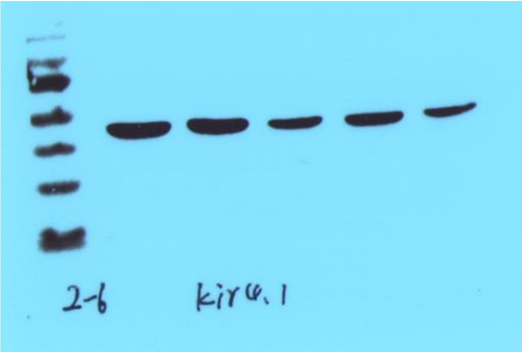

GAPDH (37 kDa)

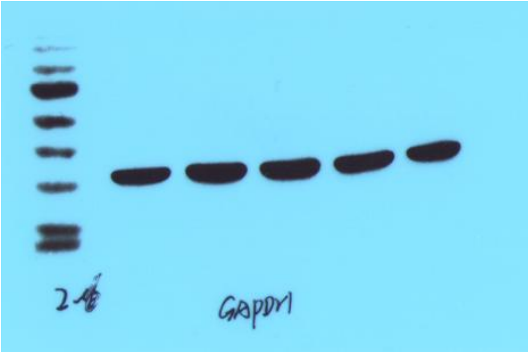

Figure 4M

Na^{+}-K^{+}-ATPase (30 kDa)

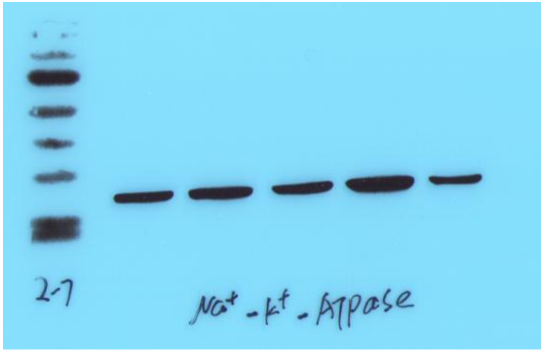

GAPDH (37 kDa)

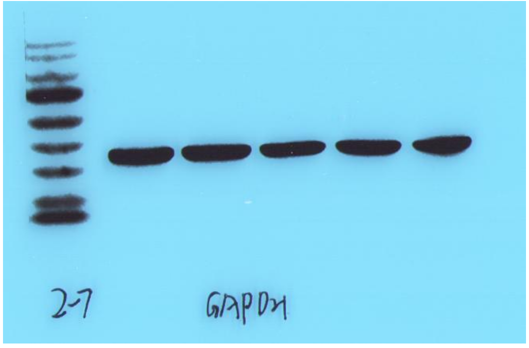

Figure 5B

TXNIP (44 kDa)

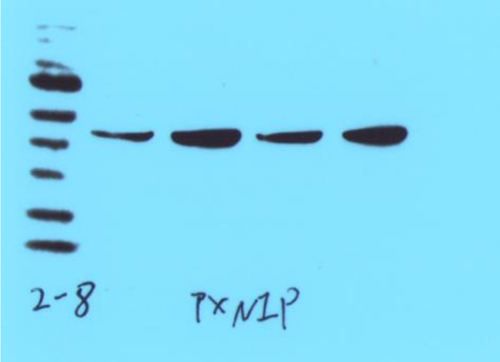

GAPDH (37 kDa)

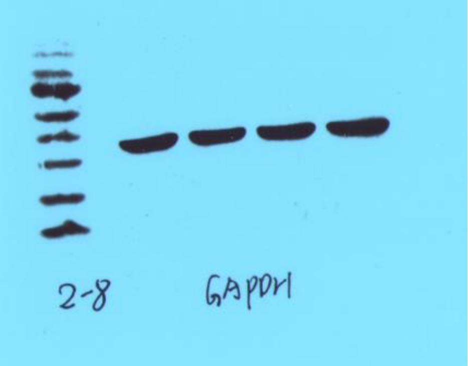

Figure 5I

GFAP (50 kDa)

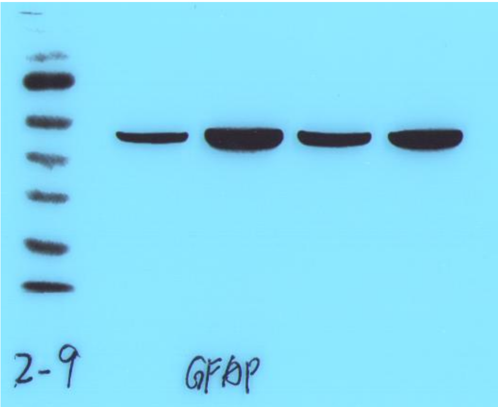

Vimentin (54 kDa)

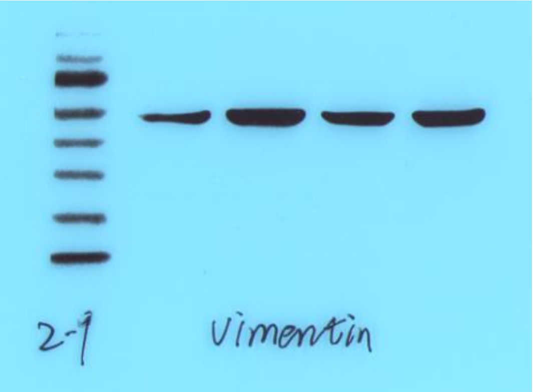

Nestin (177 kDa)

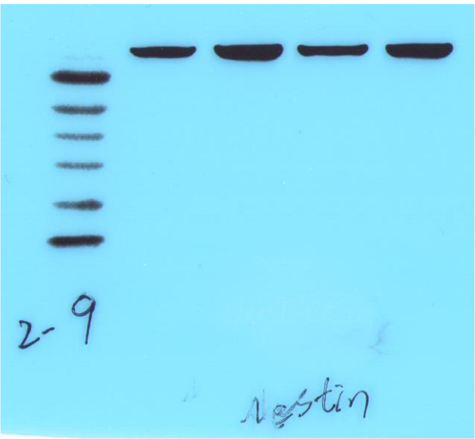

GAPDH (37 kDa)

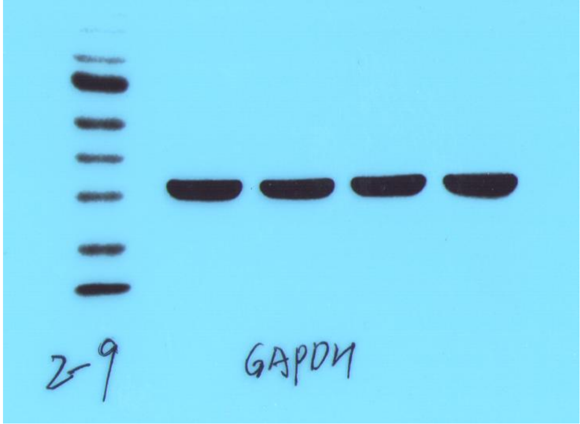

Figure 5L

Kir4.1 (42 kDa)

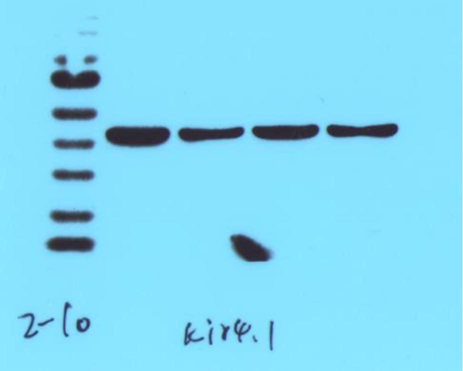

GAPDH (37 kDa)

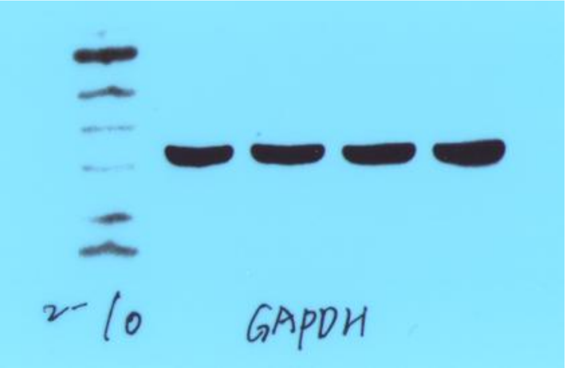

Figure 5M

Na^{+}-K^{+}-ATPase (30 kDa)

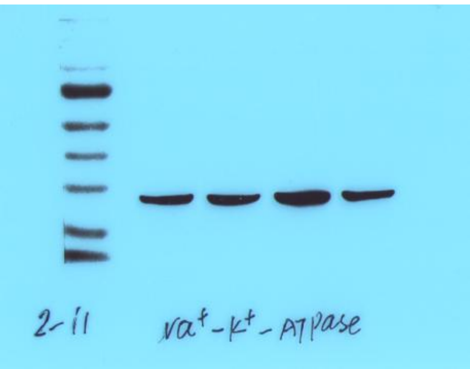

GAPDH (37 kDa)

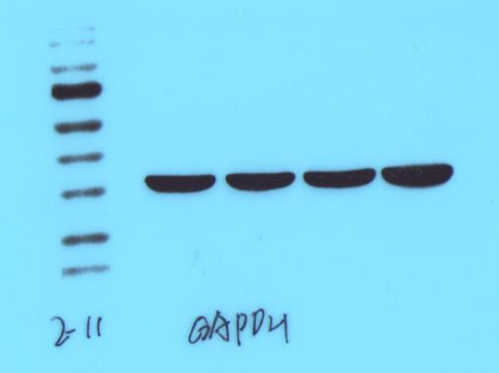

Figure 6A

p-PI3K (126 kDa)

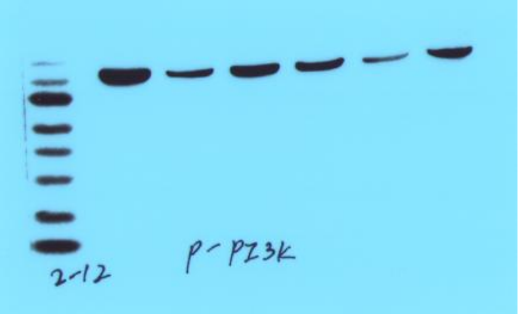

PI3K (126 kDa)

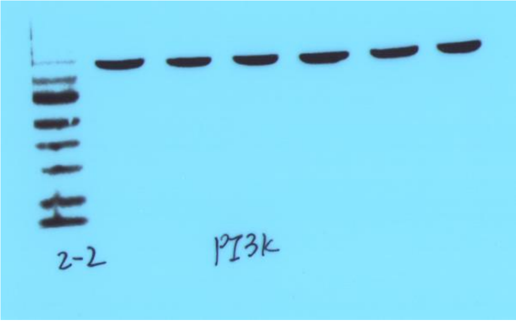

p-AKT (56 kDa)

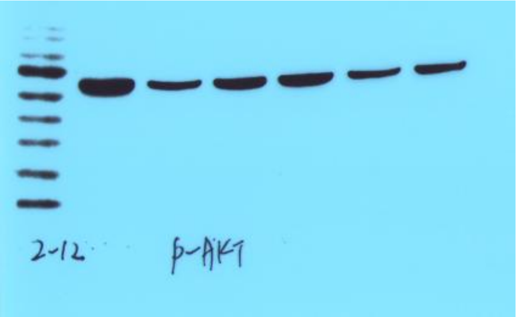

AKT (56 kDa)

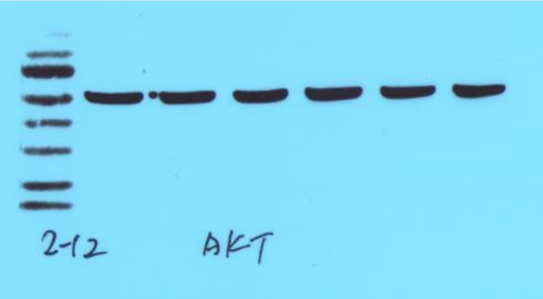

GAPDH (37 kDa)

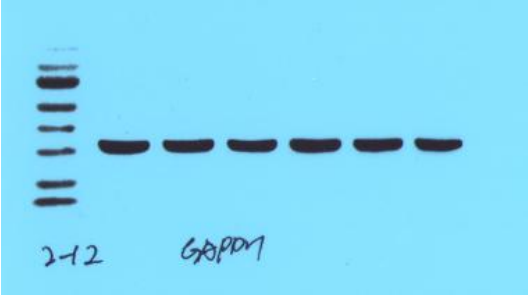

Figure 6H

GFAP (50 kDa)

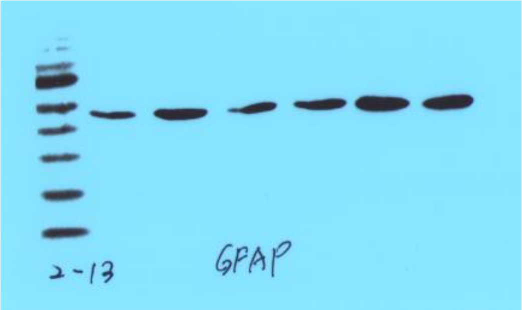

Vimentin (54 kDa)

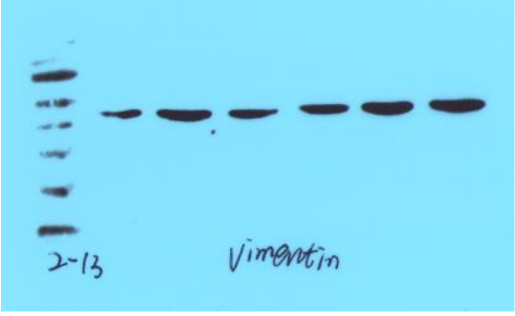

Nestin (177 kDa)

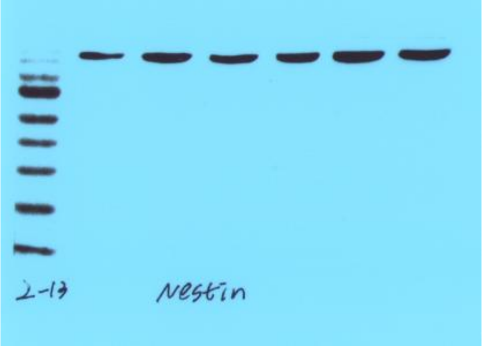

GAPDH (37 kDa)

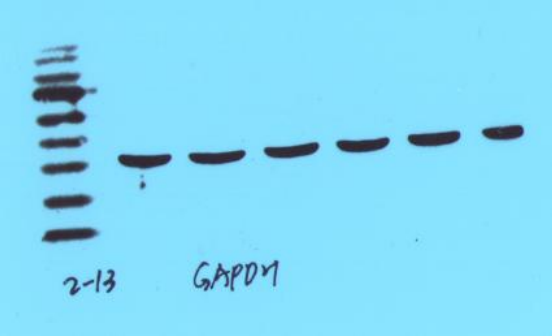

Figure 6K

Kir4.1 (42 kDa)

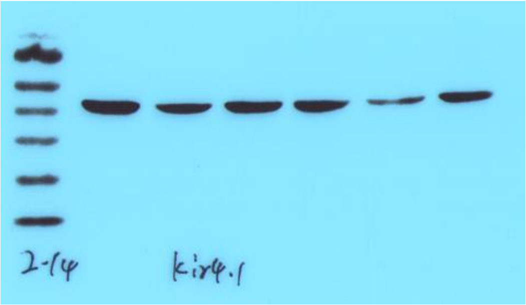

GAPDH (37 kDa)

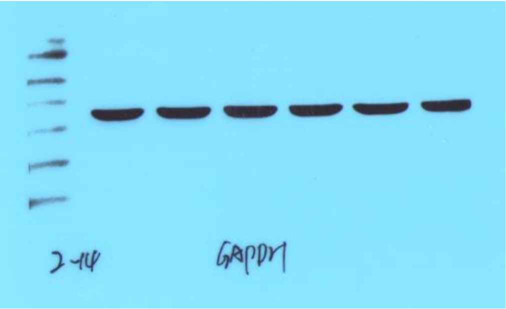

Figure 6L

Na^{+}-K^{+}-ATPase (30 kDa)

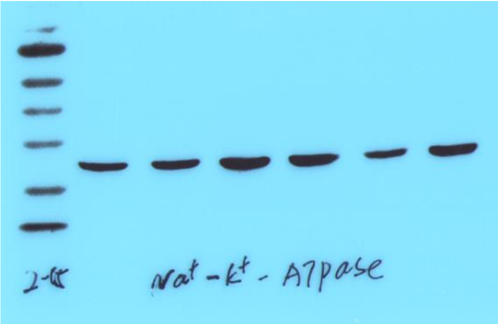

GAPDH (37 kDa)

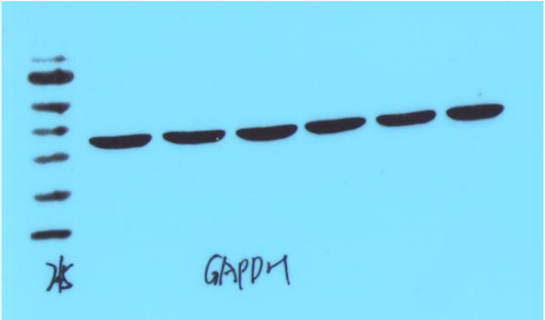

Figure 7B

TXNIP (44 kDa)

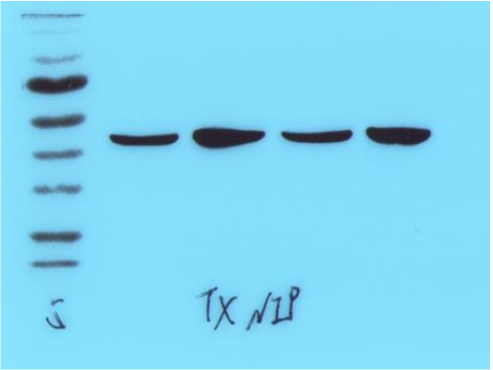

GAPDH (37 kDa)

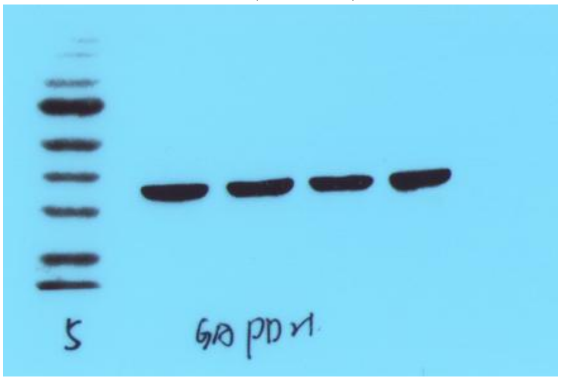

Figure 7I

GFAP (50 kDa)

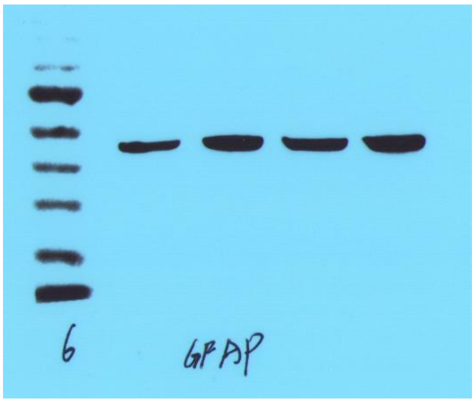

Vimentin (54 kDa)

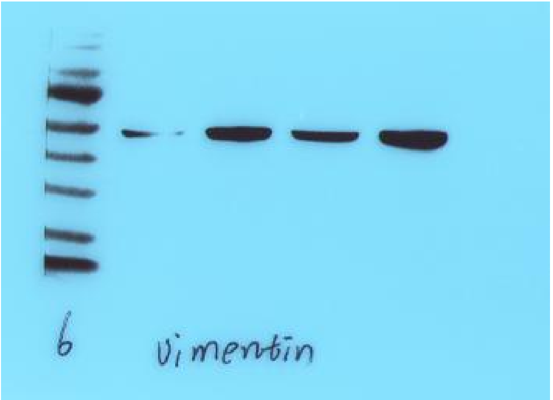

Nestin (177 kDa)

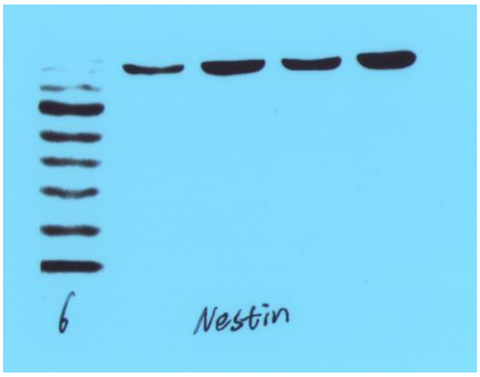

GAPDH (37 kDa)

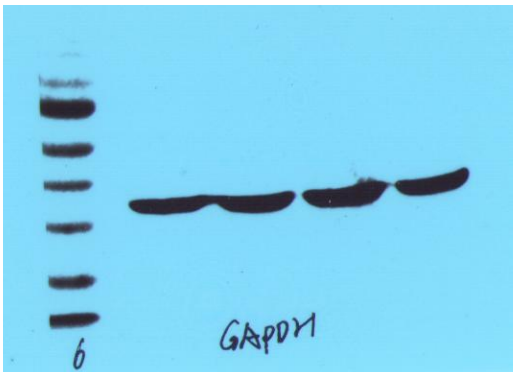

Figure 7L

Kir4.1 (42 kDa)

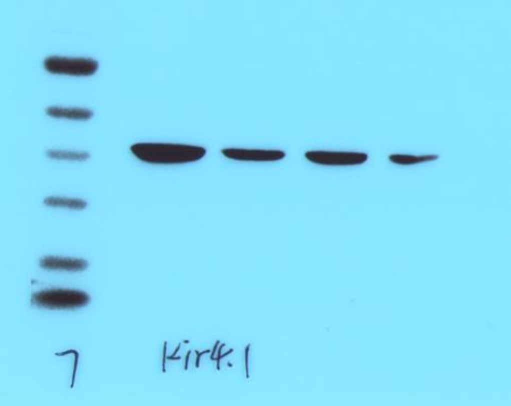

GAPDH (37 kDa)

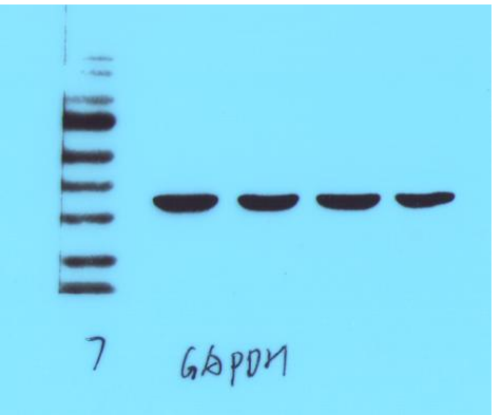

Figure 8B

TXNIP (44 kDa)

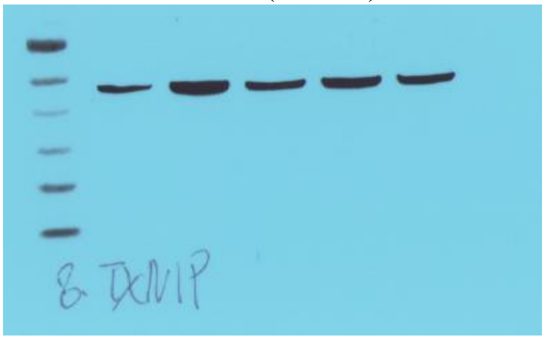

GAPDH (37 kDa)

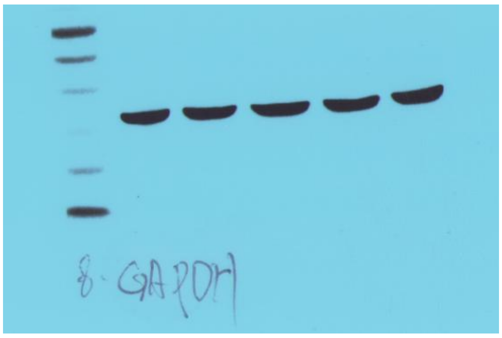

Figure 8C

p-PI3K (126 kDa)

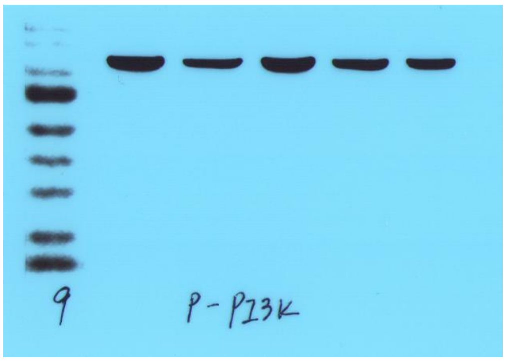

PI3K (126 kDa)

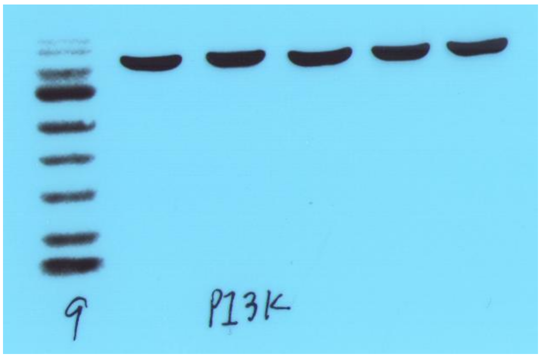

p-AKT (56 kDa)

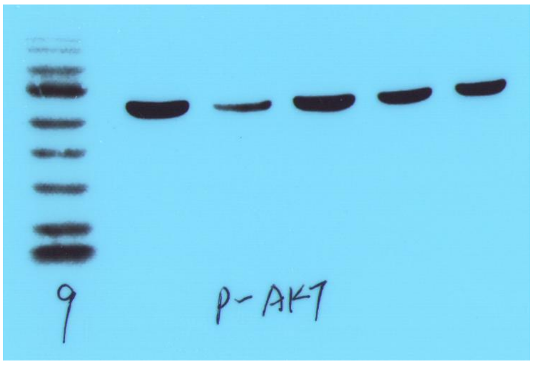

AKT (56 kDa)

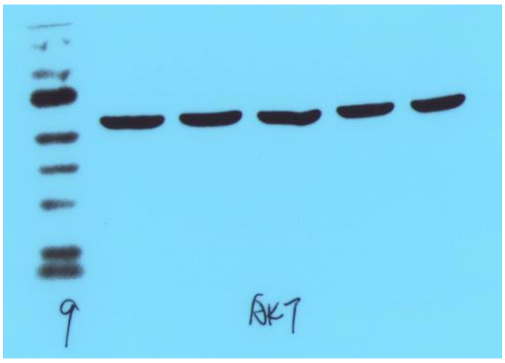

GAPDH (37 kDa)

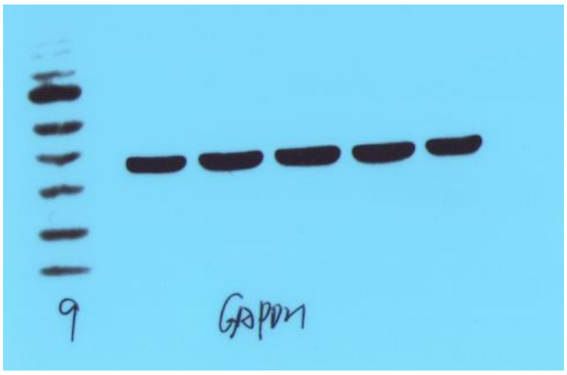

Figure 8J

GFAP (50 kDa)

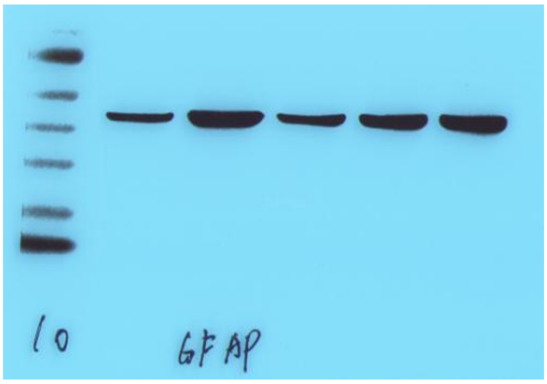

Vimentin (54 kDa)

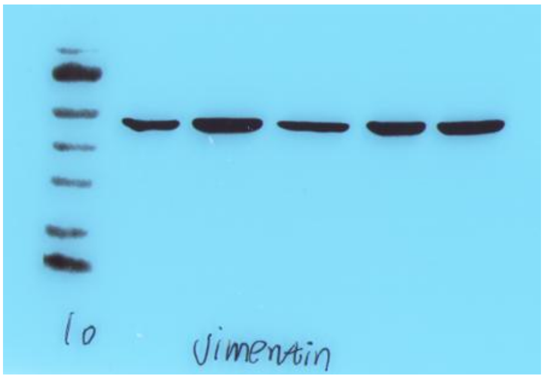

Nestin (177 kDa)

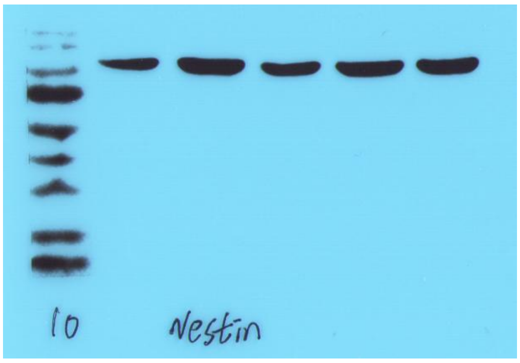

GAPDH (37 kDa)

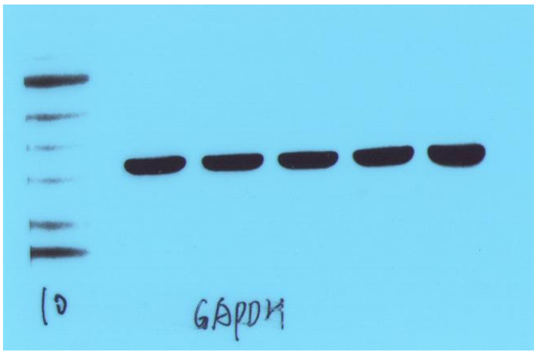

Figure 8M

Kir4.1 (42 kDa)

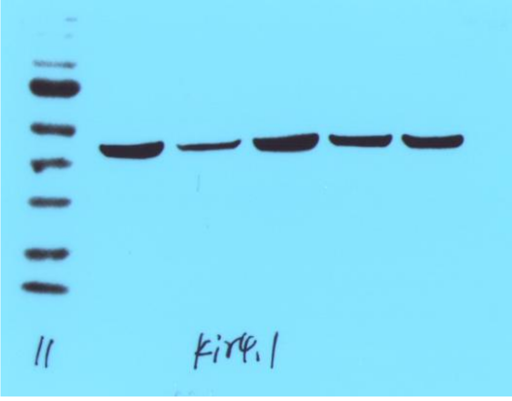

GAPDH (37 kDa)

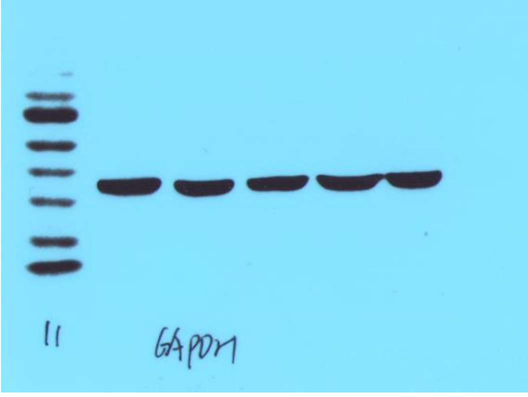

Supplement: S1 Raw image — (PDF) [file pone.0312565.s001.pdf]
